# Leveraging Partial Coherence to Enhance Nanoparticle Detection Sensitivity and Throughput in Interferometric Scattering Microscopy

**DOI:** 10.1021/acsphotonics.5c00744

**Published:** 2025-08-01

**Authors:** Chiara Lombardo, Andrea Sottini, Sarina Seiter, Gérard Colas des Francs, Jaime Ortega Arroyo, Romain Quidant

**Affiliations:** † Nanophotonic Systems Laboratory, Department of Mechanical and Process Engineering, 111842 ETH Zurich, Tannenstrasse 3, Zurich 8092, Switzerland; ‡ 27011 Université Bourgogne Europe, CNRS, Laboratoire Interdisciplinaire Carnot de Bourgogne ICB UMR 6303, Dijon F-21000, France

**Keywords:** digital holography, interferometric microscopy, iSCAT, nanophotonics, partial coherence, sensing, label-free imaging

## Abstract

Interferometric-based microscopies stand as powerful label-free approaches for monitoring and characterizing chemical reactions and heterogeneous nanoparticle systems in real time with single-particle sensitivity. Nevertheless, coherent artifacts, such as speckle and parasitic interferences, together with limited photon fluxes from spatially incoherent sources, pose an ongoing challenge in achieving both high sensitivity and throughput. In this study, we systematically characterize how partial coherence affects the signal contrast and background noise level in inline holography microscopes operated in a reflection geometry, a category that encompasses interferometric scattering microscopy (iSCAT). This approach offers a route to improve the signal-to-noise ratio in the detection of single nanoparticles (NPs), irrespective of their size and composition or the light source used. We first validate that lasers can be modified into partially coherent sources with performance matching that of spatially incoherent ones while providing higher photon fluxes. Second, we demonstrate that tuning the degree of partial coherence not only enhances the detection sensitivity of both synthetic and biological NPs but also affects how signal contrasts vary as a function of the focus position. Finally, we apply our findings to single-protein detection, confirming that these principles extend to differential imaging modalities, which deliver the highest sensitivity. Our results address a critical milestone in the detection of weakly scattering NPs in complex matrices, with wide-ranging applications in biotechnology, nanotechnology, chemical synthesis, and biosensing, ushering in a new generation of microscopes that push both the sensitivity and throughput boundaries without requiring beam scanning.

## Graphical abstract

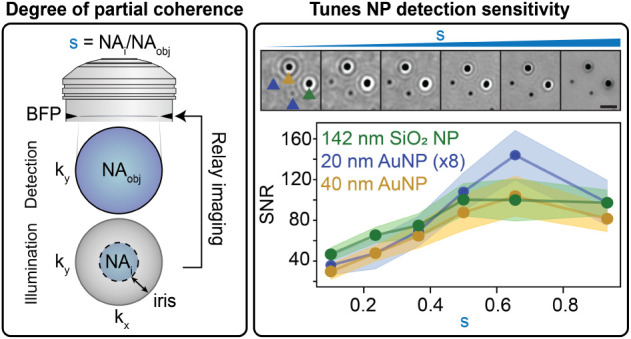

## Introduction

Recent advances in biotechnology, nanotechnology, material science, and chemical synthesis have enabled the engineering of new functional nanoparticle systems with applications ranging from gene delivery,^1,2^ targeted therapy,^3^ biosensing^4^ to heterogeneous catalysis.^5^ Additionally, molecular profiling of biological nanoparticles found in the secretome, such as extracellular vesicles (EVs), holds promise as next-generation liquid biopsies^6^ and drug delivery^7^ carriers. Consequently, there is a growing demand for high-throughput quantitative characterization tools offering single-particle sensitivity. By eliminating the need for sample handling and processing, label-free approaches are among the most suited for this task. Those based on interferometry in a reflection geometry stand out due to their inherent high sensitivity. The combination of shot-noise-limited detection with high-photon-flux light sources ensures that enough scattered photons from the smallest of nanoparticles reach the sensor and generate sufficient signal contrast, enabling single protein and nucleic acid detection, tracking, and subsequent characterization,^8–10^ as well as monitoring of complex reactions ranging from autocatalysis,^11^ nanoparticle formation^12,13^ covalent organic framework formation,^14^ and tracking of single-particle ion dynamics.^15^

The requirement for high photon flux sources is typically satisfied using high-power CW lasers, which are both spatially and temporally coherent illumination sources. However, achieving high sensitivity at high throughput with these light sources—defined here in terms of the size of the field of view (FOV) per unit time—remains a significant challenge. On the one hand, coherent artifacts such as speckles and parasitic interferences severely degrade the image quality for wide-field imaging.^16,17^ On the other hand, reducing these coherent artifacts with a spatially coherent light source (CW laser) by turning the imaging system into a partially coherent one, either by loosely focusing the beam into the BFP of the objective or opting for a confocal illumination, comes at the expense of a smaller FOV. To best capture these differences, Figure 1 illustrates the image formation principle in a reflection-based interferometric microscope for three different scenarios: (i) a coherent imaging system with a spatially coherent light source, (ii) a partially coherent imaging system with a spatially coherent light source, and (iii) a partially coherent imaging system with a spatially incoherent light source. The first case (Figure 1b, left column) is represented by Köhler illumination, where larger FOVs result from focusing the light source tightly into the BFP of the objective, essentially lowering the spatial frequency bandwidth, which in turn increases the spatial coherence of the imaging system, thus leading to coherent artifacts and lower spatial resolution, as represented in the optical transfer function (OTF). The second case (Figure 1b, middle column) represents a partially coherent microscope resulting from increasing the size of the illuminating beam at the BFP, which can be experimentally achieved by weakly focusing a laser into the BFP or confocally illuminating the sample. Under this scenario, the spatial frequency bandwidth increases, thus lowering the spatial coherence and increasing the resolution of the imaging system. However, this reduces the FOV, as shown in the middle column of Figure 1b. Solutions to extend the FOV exist, either in the form of rapid beam scanning of a weakly focused beam with acousto-optic beam deflectors,^18^ raster scanning confocal detection,^19,20^ spinning disk confocal,^21^ or rotational integration of oblique scanning^22^ yet these come with drawbacks such as high peak intensities and limited scanning speeds that may ultimately restrict the throughput. The third case showcases an alternative solution to this problem, which involves using a spatially incoherent illumination source,^23–27^ such as multimode fiber-coupled light-emitting diodes (LEDs), which not only deliver larger FOVs with minimal coherent artifacts but also flat-top illumination profiles^28^ (Figure 1b right column). Similarly, the use of rotating diffusers applied to inline holography in reflection geometry was also recently demonstrated for the first time alongside LEDs^26^ and later showed that, when combined with a tunable lens, it can be used to further tailor the spatial coherence of the light source.^29^

**Figure 1:**
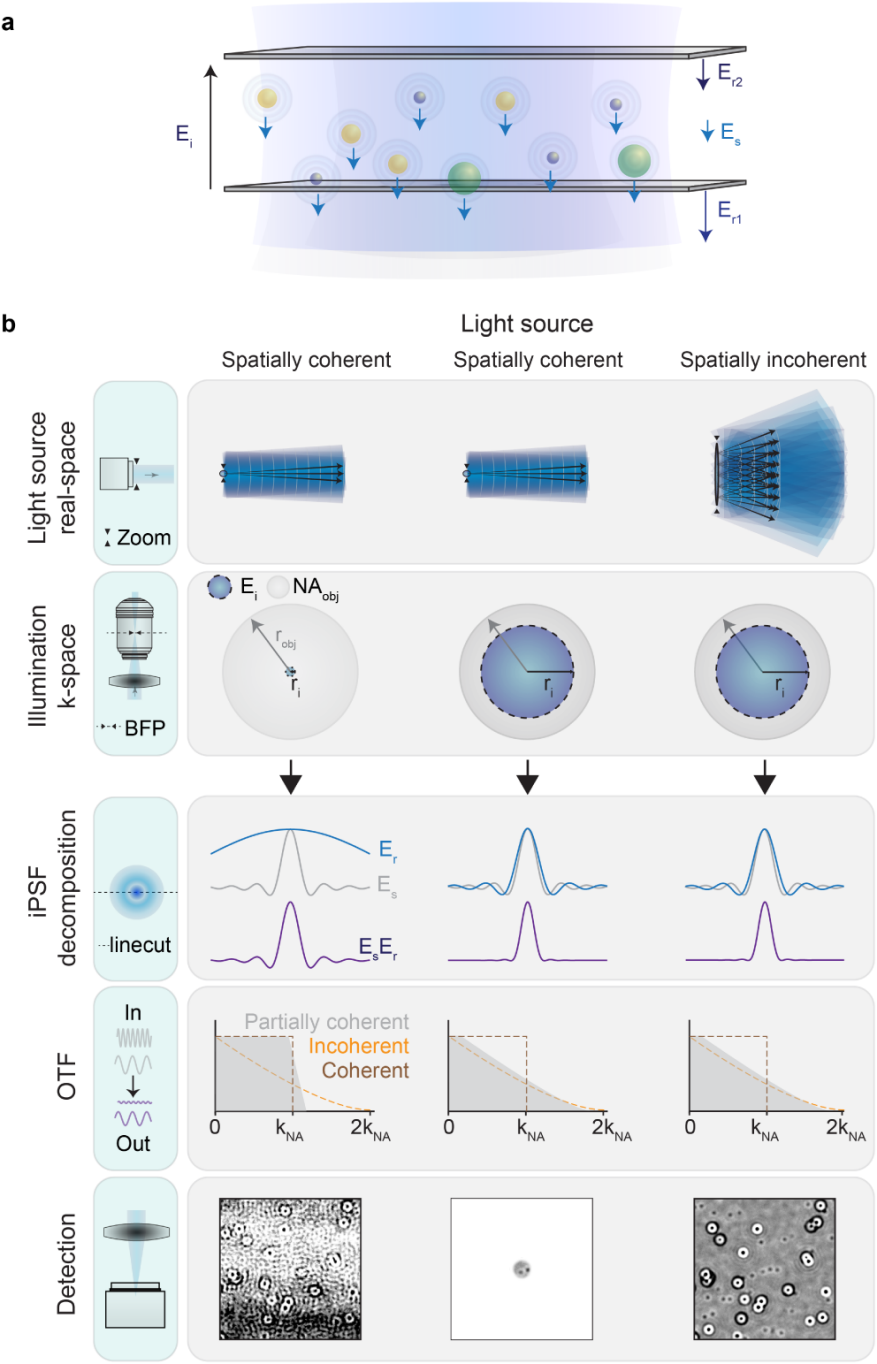
(a) Cartoon depicting the principle of interferometric detection of nanoparticles in a reflection-based geometry within an imaging chamber containing two interfaces, representing a common sample configuration encountered in many flow cell or microfluidic chip designs. Arrows represent the different electric field contributions. (b) Image formation process in partially coherent interferometric systems for different light sources and degrees of spatial coherence. The image frames show a zoomed-in area of approximately 10 × 10  μm^2^ from the total sample illuminated, and the contrast range is restricted to ±0.07. E_*i*_: incident electric field, E*s*: scattering electric field, E_*r*1_: reflection electric field from the bottom glass/water interface, E_*r*2_: reflection electric field from the top water/glass interface, BFP: objective back focal plane, NA_obj_: numerical aperture of the objective, r_obj_: aperture size at the back focal plane of the objective, **r**_obj_: illumination beam size at the back focal plane of the objective, iPSF: interferometric point spread function, OTF: optical transfer function, k_NA_: spatial frequency corresponding to the numerical aperture of the detection objective.

Although decreasing spatial rather than temporal coherence is more effective in reducing PSF ringing, parasitic interferences, and speckle contrast in inline holography,^16^ the coherence of the imaging system also plays a significant role in the detection of NPs. Specifically, coherence modulates the detected scattering signal contrasts from single NPs, as well as the phase transfer function^30,31^ thereby determining how their detection sensitivity varies as a function of sample focus position. Furthermore, upon implementing partially coherent systems in a reflection geometry, the contrast signal generated by individual scatterers is influenced by the experimental configuration of the system, ranging from the strength of the reference electric field given by the Fresnel coefficient from the glass/imaging medium interface, the scattering emission profile at said interface, the imaging resolution, and the presence of parasitic backreflections. As a result, there have been discrepancies in the trends that describe how the NP contrast signals vary with the degree of partial coherence. These discrepancies largely stem from differences in the experimental implementation of the partially coherent imaging system, with some groups reporting the highest signal contrast by reducing the spatial coherence when using an LED,^24^ while others showing the opposite trend when the light source is relay-imaged into k-space, i.e., the back-focal plane (BFP) of either a low NA^32^ or high NA objective.^27,29^

Given the discrepancies between groups and the general knowledge that partial coherent imaging systems should be preferred when high-throughput imaging weakly scattering objects, the question of how to tailor the degree of partial coherence to maximize the SNR and quantify single-particle signals in the least amount of time remains largely unanswered. This study addresses this gap and provides a solution applicable to metallic, dielectric, and biological nanoparticles for interferometric microscopes in a reflection geometry. To do so, we developed a platform that allows the simultaneous tuning and measurement of the degree of partial coherence. We specifically characterized the dependence of the signal contrasts and the background noise contributions to find the experimental parameters that not only optimize the SNR and acquisition throughput but also reliably detect all NPs within the same focus position. We further demonstrated that such partially coherent systems are compatible with a differential imaging modality, thus enabling sensitivities compatible with single protein detection but with orders of magnitude larger FOVs compared to the state-of-the-art. The results from this work show that partially coherent systems provide a route to pushing the sensitivity and throughput of state-of-the-art interference-based label-free microscopes to new boundaries.

## Results and Discussion

### Working Principle and Experimental Implementation

In this work, we quantitatively assessed how spatial coherence—in other words, the degree of partial coherence—influences the detection sensitivity at the single-particle level. To achieve this, we incorporated a module that precisely controls and measures this parameter into an existing reflection-based interferometric microscope. The degree of partial coherence was quantified by the coherence parameter, *s*, defined as the ratio of the numerical aperture of illumination to that of the detection objective: *s* = NA_*i*_/NA_obj_. This parameter extensively used in microscopy and holography^33^ to describe the spatial coherence of the imaging system, on the one hand, allows classification of the imaging system into coherent for s→0, partially coherent for 0 < *s* < 1, and incoherent for *s* ≥ 1, and on the other hand, provides a framework to characterize partially coherent systems from the perspective of spatial frequency bandwidth.^16^ To tune *s* experimentally, we varied the size of an adjustable iris relay imaged to the back focal plane (BFP) of the objective, effectively decoupling NA_*i*_ from NA_obj_ (Figure 2a). As light sources, we used both a laser diode and an LED, with their respective spectra shown in Figure 2b. In addition, light from the laser diode was turned into a partially coherent illumination source by focusing it onto a rotating ground glass diffuser (RGG)^34^ before coupling it into a multimode fiber (Figure 2c).

**Figure 2:**
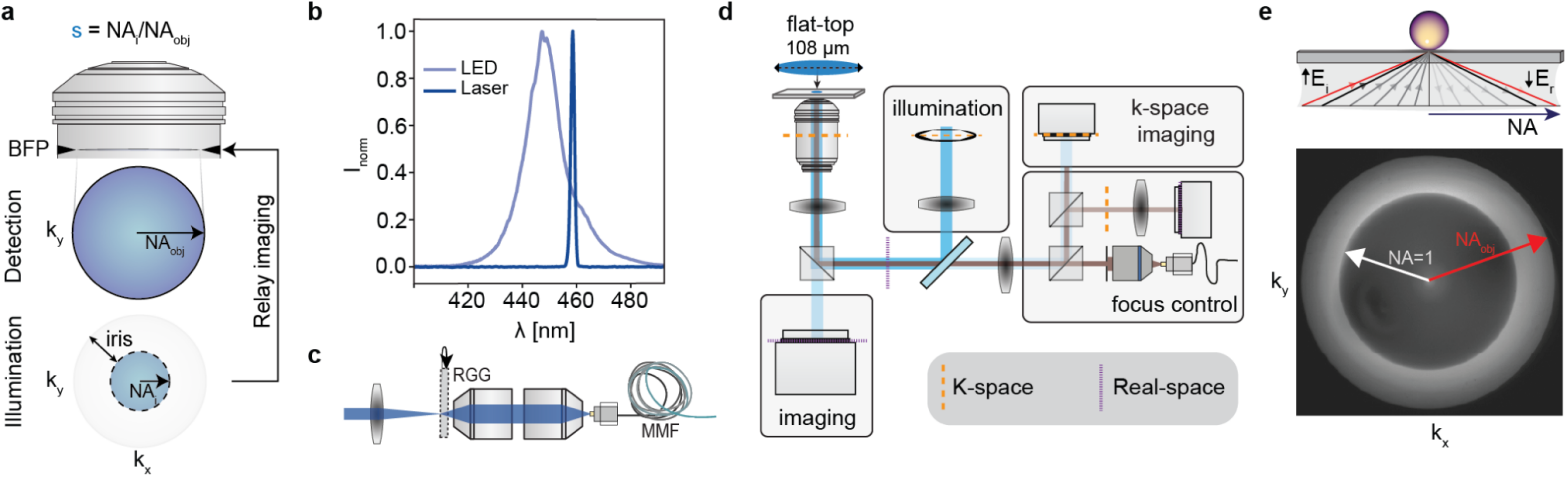
Working principle and experimental implementation. (a) Cartoon depicting the tuning of the degree of partial coherence. (b) Spectra of the two sources used for illumination: LED and laser diode. (c) Scheme for turning a laser into a spatially incoherent light source using a rotating diffuser and multimode fiber. (d) Experimental setup schematic with different lines indicating the locations of the real and k-spaces. (e) Representative k-space image of a glass/air interface for measuring NA_*i*_. Image taken for a fully open adjustable iris.

Figure 2d depicts the experimental setup comprising a custom-built common-path interferometric microscope operating in reflection mode, featuring four different modules: imaging, focus stabilization, light source input, and measurement of the NA_*i*_. The focus control module, together with the XY motorized sample stage, enabled automation of the XYZ sample scanning assays with focus position stabilization to within 10 nm. The NA_*i*_ was tuned in the illumination module by adjusting the size of the iris and subsequently measured in a separate k-space imaging channel. Figure 2e shows a representative k-space image for a sample composed of immobilized nanoparticles at the glass–air interface. The observed bright ring corresponds to the total internally reflected incident angles of illumination (NA_*i*_ ≥ 1), with the inner and outer radii of the ring corresponding to NA = 1 and NA_obj_, respectively. Using the value of NA = 1 from the glass–air interface as an internal reference^35^ we determined all input NA_*i*_ and, therefore, *s*.

### Comparison between Spatially Incoherent Light Sources for a Partially Coherent Microscope

One of the main advantages of using LEDs in partially coherent imaging systems is the simultaneous reduction of speckle noise and access to large FOVs. However, their lower photon flux compared to lasers restricts the available photon budget for either sensitivity or throughput, but not both. To overcome this limitation, we converted a laser into a spatially incoherent light source by increasing its spatial frequency bandwidth when illuminating through an RGG and coupling the transmitted light into a multimode fiber with more than 50% efficiency (see Methods). This effectively suppresses coherent imaging artifacts associated with the laser via angular, spatial, and temporal domain averaging.

To validate the equivalence between the two light sources at the same fluences, irrespective of particle size and refractive index, we evaluated the particle contrast and image noise from a polydisperse sample containing 20 nm Au, 40 nm Au, and 142 nm SiO_2_ nanoparticles at a fixed partial coherence parameter *s* = 0.51 (NA_*i*_ = 0.73). To increase particle statistics, the sample was raster-scanned over a total area of roughly 450 × 450 μm^2^, corresponding to *N* = 10 FOVs of approximately 45× 45  μm^2^. Notably, the sensor limited the size of the FOV recorded from a single image, given the nearly 4.5× larger illuminated area (9331 μm^2^). To ensure that the optimal signal contrast for each NP type was recorded, at each sample position, the focus was scanned across 3 μm in 100 nm steps, denoted here as a defocus scan. Figure 3a shows representative images of the polydisperse sample deposited on the glass coverslip at two different focus positions.

**Figure 3:**
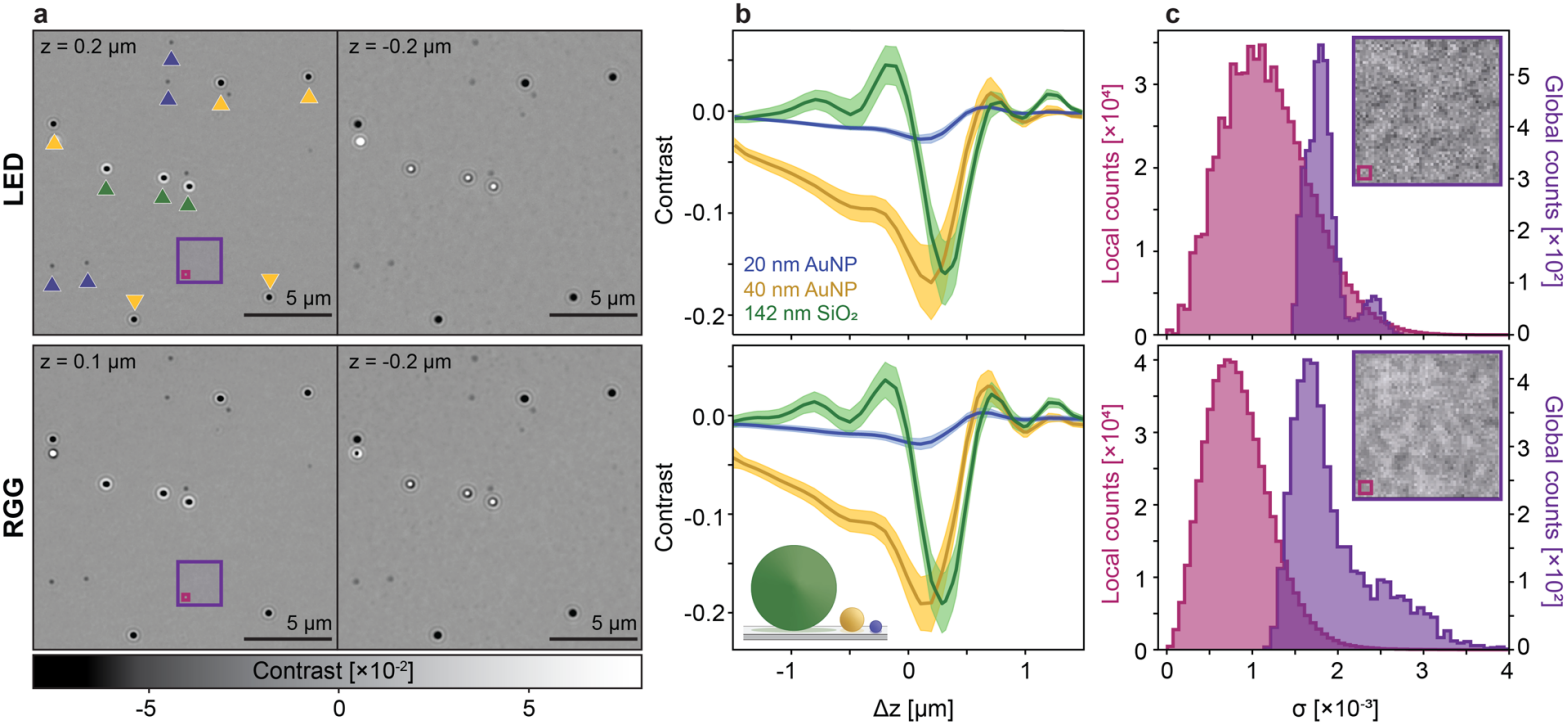
Effect of light source for delivering partial coherence. (a) Representative images of the same sample region area at two different focus positions illuminated with two different spatially incoherent sources at a fixed *s* = 0.51, NA_*i*_ = 0.73. The polydisperse NP sample consists of 20 nm AuNP, 40 nm AuNP, and 142 SiO_2_ NP sample. (b) Corresponding distribution of particle contrasts as a function of focus position. Each color represents a different particle population, with solid lines representing the mean, and the shaded regions representing ± one standard deviation. The number of detected nanoparticles consisted of 20 nm AuNP (241), 40 nm AuNP (136), and SiO_2_NPs (31) for the measurement performed using the LED. For the RGG experiment, the number of detected nanoparticles consisted of 20 nm AuNP (285), 40 nm AuNP (105), and SiO_2_NPs (51). (c) Local (pink) and global (purple) background noise distributions measured in standard deviations (σ) for different light sources. The corner images depict an empty region of the sample, highlighted by the red box in (a), with the contrast adjusted to ± 0.01 to emphasize the background noise. The areas within the image indicate the regions used to calculate global and local standard deviations for the histograms, with one standard deviation computed per region. The pink area includes 51 × 51 pixels^2^ and the purple area includes 3 × 3 pixels^2^, with one pixel corresponding to an area of 45 × 45 nm^2^.

To characterize the particle contrast as a function of defocus, individual particles were localized and subsequently classified. Defocus scans of samples containing only one particle species at a time served as a reference for classification. Figure 3b shows the average contrast curves ± one standard deviation (shaded area) for each particle population. The shaded regions reflect the intrinsic size dispersion of each particle distribution. These scans showed a characteristic oscillatory behavior between positive and negative contrast, a pattern that was distinct for each of the three particle species. Furthermore, the maximum contrast magnitude for each particle type occurred at different defocus positions, consistent with contrast tuning with the Gouy phase^9,36^ and the phase transfer function.^30,31^ Minor deviations between contrast curves from the two different light sources were attributed to slight spectral differences. Nonetheless, these defocus scans demonstrated the equivalence between both illumination schemes and the potential to use these scans as particle classifiers.

To compare these two illumination schemes with respect to the background noise, we analyzed two noise metrics corresponding to the local and global fluctuations within each image. Local noise fluctuations quantified the shot noise within the image, whereas global noise fluctuations predominantly measured the speckle and background roughness contributions. As a first step, we segmented all pixels within an image corresponding to the background, i.e., excluding those counted as particles. Local background noise was computed as the standard deviation within a 3 × 3 background pixel area. The choice of an interrogation area significantly smaller than the diffraction limit minimized any speckle or substrate roughness contributions. Global noise was calculated as the standard deviation within a 51 × 51 background pixel area. Figure 3c shows the distribution of local (pink) and global (purple) background noise from all frames under the two illumination schemes, with the global background noise at least 2-fold higher than the local one. As expected from a shot-noise-limited measurement, both partially coherent schemes showed comparable local background noise levels when illuminated at similar fluences. Similarly, speckle and substrate roughness contributions increased the noise level above shot noise, with slightly higher values for the RGG-based illumination due to the presence of low spatial frequency components that had not been effectively suppressed during the integration time of the sensor.

In summary, for a partially coherent microscope, lasers combined with an RGG can perform just as well as LEDs and additionally deliver higher fluences. This enables imaging of larger fields of view at higher temporal resolutions, thereby increasing the overall throughput of the imaging system. Given the equivalence of the two light sources and the fact that LEDs have already been successfully implemented to measure biological NPs such as EVs and single proteins,^26^ all subsequent results were performed with an RGG-based laser as the illumination source.

### Effect of Partial Coherence on the Signal-to-Noise Ratio for Particle Detection

To determine how the degree of partial coherence affects particle detection SNR, we repeated the defocus scan of the polydisperse sample under different coherence parameters, *s*, by varying the NA_*i*_ but keeping the detection NA fixed and imaging the exact same sample area. Figure 4a shows representative zoomed-in regions containing all three NP species with their respective ensemble defocus scan contrast curves. For these representative images, we chose the focus position at each parameter *s* that maximized the contrast for the smallest particles (20 nm AuNP), as these were not the same for all particles or for different NA_*i*_. Specifically, the focus position of the maximum negative contrast, indicated by the dotted vertical lines in Figure 4a, shifted to higher defocus positions as the degree of partial coherence increased. Similarly, the amplitude of contrast oscillations in the defocus curves decayed with increasing *s*. To further validate our experimental data, we developed an imaging model for the detection of NPs as a function of defocus for partially coherent systems based on interferometric detection in reflection geometry (see section S1 and Figures S1–S3). For all three NPs, the model and experiment showed excellent agreement.

**Figure 4:**
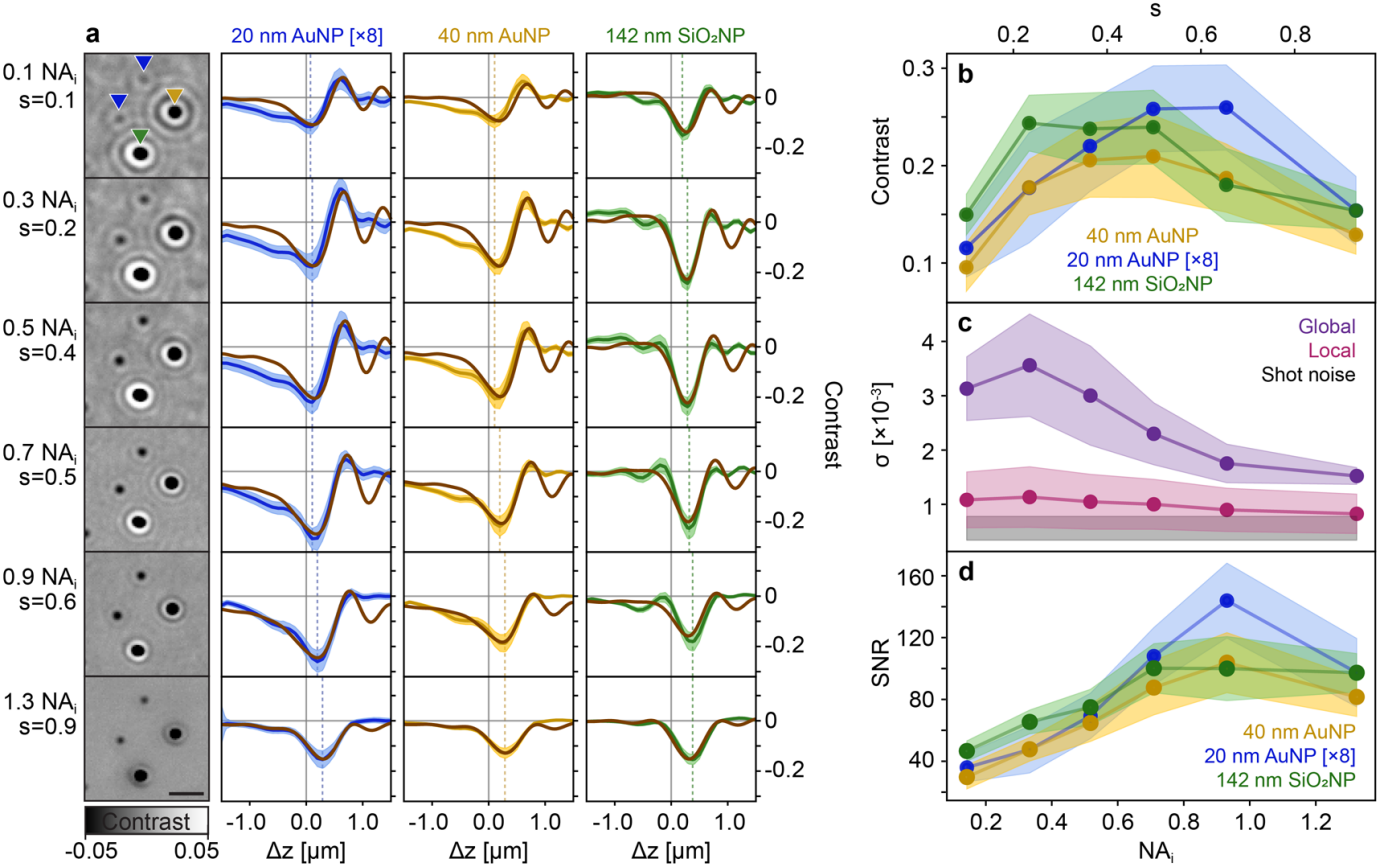
Effect of partial coherence on single NP detection. (a) Left: Inline holography images of two 20 nm AuNPs (blue triangle), a 40 nm AuNP (orange triangle), and a 142 nm SiO_2_ (green triangle). The images were acquired at the focus position where the smallest constituents, 20 nm AuNPs, exhibit the largest contrast, as indicated by the blue vertical dashed lines in the right plots. Scale bars: 1 μm. Right: Experimental vs simulation population-averaged contrast defocus curves for each NP species. Colored solid lines (blue, orange, green) and shaded regions indicate the experimental average and plus/minus one standard deviation, whereas brown curves correspond to simulation data (see S1). Increasing from the smallest to the largest NA_*i*_, the particle counts of each NP species are: 20 nm AuNPs (420, 428,404, 424, 249, 291), 40 nm AuNPs (249, 214,245, 183, 203, 179), and SiO_2_ NPs (452, 1302,1601, 1409, 1582,1828). (b) Population-averaged values of maximum absolute contrast plotted against the degree of spatial coherence. (c) Local and global background noise as a function of the degree of spatial coherence. The black region indicates the shot noise limit. (d) Population-averaged SNR as a function of the degree of spatial coherence, retrieved by dividing the population-averaged contrast shown in (b) by the global noise shown in (c).

As expected from partially coherent imaging systems, as *s* (NA_*i*_) increased, the optical resolution also increased, while the speckle contrast, PSF ringing, and substrate roughness contributions—mostly composed of high spatial frequency components—decreased. In addition, the signal contrast for each particle increased as a function of *s*, before dropping slightly as NA_*i*_ approached the value of the refractive index of the solution. We attribute this latter drop in contrast to the increase in effective reflectivity associated with including total internal reflection contributions (see S2.1 and Figure S4).

To evaluate how the increase in particle contrast, together with the reduction in background noise with increasing *s* values, translates to particle detection SNR, we first isolated each contribution individually. Figure 4b shows the average contrast magnitude for all three NP species, demonstrating at least a 2-fold enhancement compared to the lowest *s* value evaluated. Notably, the smaller the particle, the higher the contrast enhancement and its occurrence at higher degrees of partial coherence. We partially attribute this trend to the increase in resolution (see S2.2).

For the noise component, we compared both the local and global background noise metrics (Figure 4c). The local background noise remained constant, as expected from illumination at similar fluences for the different partially coherent systems, with values within the range of a shot-noise-limited measurement based solely on camera counts. In contrast, the global noise level, which includes signals assigned to glass roughness contributions, decreased approximately 2-fold as a result of the reduction of coherent artifacts. We emphasize that the reduction in noise was independent of NP sample type and defocus position. Moreover, the reduction in noise also indicated that much of the signals attributed to the glass roughness contributions largely stem from the coherent superposition of scattering signals, i.e., from a strongly speckle-dominated signal.

We then computed the SNR as the ratio of the population average particle contrast magnitude to the global background noise (Figure 4d). The overall trend showed that the SNR can be increased between 2- and 4-fold within the range of partial coherence parameters tested, with an optimal window within 0.7 < NA_*i*_ < 1.3. It should be emphasized that these enhancement factors underestimate the true enhancement relative to the coherent case, typically associated with widefield interferometric scattering microscopy (with *s* ≪ 0.1). This is simply because coherent artifacts degraded the image so severely (Figure 1, representative image, bottom left corner) that any quantitative particle characterization was intractable.

### Partial Coherent Detection Applied to Biological Nanoparticles

Next, we repeated the defocus scans as a function of *s* on a sample containing H358 cell culture-derived EVs (Figure S7) to determine whether similar SNR enhancements are expected with biological NPs. Here, the intrinsic size and refractive index heterogeneity of EVs make them an ideal system for observing general trends that extend to other biological NPs, especially relevant when no a priori information is available for each NP. Specifically, nanoparticle tracking analysis measured an average population diameter of 124 ± 57 nm (see Figure S7). Based on their size- and cargo-dependent refractive index (1.36–1.40)^37^ as well as physical dimensions, the optical response of a fraction of EVs is expected to show similarities to the 142 nm SiO_2_ NP. However, the intrinsic size and refractive index heterogeneity of EVs limits precise contrast predictions. Figure 5a plots the SNR distribution of all single EVs detected for varying coherence parameters, with the dashed-dotted lines on each distribution indicating the 95th percentile. For the SNR calculation, we assumed that the optimal contrast had a negative value for all particles. Similarly to synthetic NP assays, increasing *s* led to SNR enhancements for the EV sample, with the maximum occurring in the range of 0.7 < NA_*i*_ < 1.3. From an ensemble perspective, an almost 4-fold enhancement was observed, with the contrast enhancement contributing more than 60% to this increase (Figure S8). Nevertheless, we must point out that this metric corresponded to a lower bound, as many EVs were not detected at lower *s* values simply because their SNR fell below the detection threshold.

**Figure 5:**
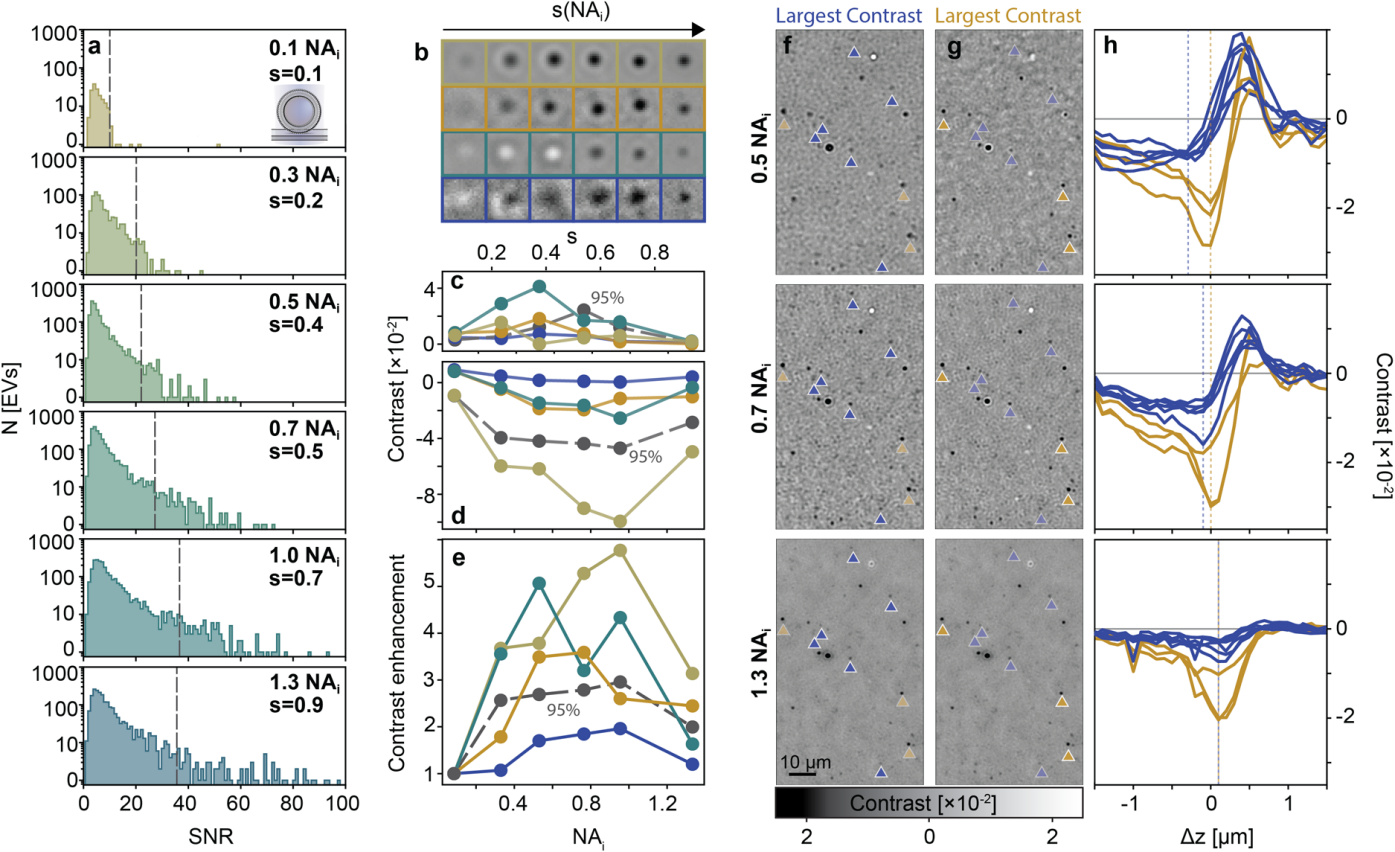
Effect of partial coherence on biological nanoparticle detection. (a) Distribution of the maximum SNR of all detected EVs as a function of the degree of partial coherence. The dashed gray line indicates the 95th percentile of the population. Increasing from the smallest to the largest NA_*i*_, the counts of considered EVs are: 160, 683, 1746, 2501, 2491, 2098. (b) Examples of four EVs sampled at different illumination NAs. Each cutout shows the axial position at which the given EV reaches its maximum contrast. (c) Maximum positive contrast values of the EVs shown in (b), along with the 95th percentile of the full EV population. (d) Maximum negative contrast values of the same EVs, along with the 95th percentile of the population. (e) Enhancement of the absolute contrast of the four selected EVs as well as the 95th percentile of the population. (f-g) Zoomed-in images from the defocus scan at three selected coherence parameters. The axial positions were chosen to maximize the contrast of the EVs indicated by the blue arrow (f) and yellow arrow (g), respectively. (h) Contrast-defocus curves of the EVs marked in (f) and (g) at their respective NA_*i*_. The vertical dashed lines indicate the axial positions at which the images in (f) and (g) were acquired.

To better estimate the range of SNR enhancement in this highly heterogeneous NP system, we examined how the contrast varies as a function of *s* at the single-particle level, given that the noise contributions remain invariant across particle types. Figure 5b shows four EVs, with each zoomed-in image along a row corresponding to the focus position that optimizes the signal contrast for a given degree of partial coherence. These four EVs were selected on the basis of exhibiting low, intermediate, and high contrast values as a way to indirectly show the heterogeneity in possible size and refractive index within the full population of EVs. In contrast to synthetic NPs (Figure 4b), EVs displayed a more complex contrast dependence on *s*, including contrast inversions for some particles. Here, we denote contrast inversions as a switch in the sign of the largest magnitude signal extracted from a defocus curve as a function of tuning the degree of partial coherence, not to be confused with signal inversions occurring within the contrast defocus scans. To monitor this contrast inversion, Figure 5c,d shows the maximum positive and minimum negative contrast values for each EV, extracted from their respective contrast defocus curves (Figure S9), with the dotted line marking the 95th percentile of the ensemble. Except for the EV with the largest contrast magnitude (olive line), all others underwent a signal contrast inversion: positive at low *s* and negative at high *s*. This highlights the complex role both the Gouy phase and the phase transfer function play in modulating signal contrast at lower degrees of partial coherence. Finally, we computed the enhancement as the absolute value of the optimal contrast at each *s*, normalized against the optimal contrast at the lowest tested *s*, with values ranging within 2- and 6-fold increase in the magnitude of the signal contrast (Figure 5e). Once again, these values underestimate the overall enhancement relative to the coherent case, because of the highly detrimental coherent artifacts. Nevertheless, these observations indicate that optimal results were obtained for partially coherent systems within 0.7 < NA_*i*_ < 1.3, not only because of the overall contrast enhancement and accompanying reduction in background noise fluctuations, but also because of their consistent sign of the contrast signal, thus reducing ambiguity within the choice of focus position to optimally image at.

A crucial step in single-particle-based sensing applications aimed at heterogeneous samples is the detection and subsequent characterization of NP contrast signals, which are often only taken at a single focus position. However, as shown in Figure 4b focus positions that maximize such signals strongly depend on both the particle properties and the *s* parameter. To illustrate how critical this scenario is in low-*s* imaging systems, Figure 5f,g shows representative images of EVs immobilized on the surface, taken at two different focus positions obtained from a defocus scan, alongside the contrast defocus curves for a subset of EVs (Figure 5h). Each focus position optimizes the contrast of a particular subset of EVs, indicated by blue (Figure 5f) or orange (Figure 5g) arrows. Note that for the measurements performed at NA_*i*_ = 0.5, when the contrast magnitude of the subset marked in orange was maximized, the contrast of the subset marked in blue approached a zero crossing, causing the SNR of some of these EVs to fall below the detection threshold. This experiment serves as an example that interpreting particle contrasts from experiments performed at a single focus position warrants caution, as the measured contrast may not be representative of a given particle species. However, as *s* increased, this effect significantly reduced as the distance between the two focus positions decreased and eventually converged to within 10 nm.

These results lead to two different approaches for characterizing heterogeneous NP samples that depend on the degree of partial coherence. If the degree of partial coherence is low, using a single focus position may severely underestimate both the count and contrast of entire subpopulations of NPs. Instead, either the system PSF should be engineered^38^ to make it insensitive to defocus, or defocus scans should be measured, the latter offering potential advantages in the form of richer information content that can be used as particle classifiers.^39^ If the degree of partial coherence is high, a single focus position may suffice, as the information content about the particle properties within a contrast defocus decreases, with all particle types tested tending to converge to a similar shape.

### Compatibility with Differential Imaging: Single Protein Sensitivity

Finally, most interferometric-based microscopies leverage the intrinsic shot noise-limited nature of detection through differential imaging in the absence of sample drifts, whereby a continuously updated background is subtracted from an ongoing set of images, combined with frame averaging, to drastically improve the sensitivity limits of a single-shot acquisition. To confirm that tuning the partial coherence also enhances the SNR under this imaging modality, we chose a test assay ubiquitously encountered in label-free protein detection and mass photometry: quantification of the nonspecific binding of single proteins onto a glass coverslip (Figure 6a). For this assay, we used human thyroglobulin (TG), a 660 kDa dimeric protein that spans 28 × 20 × 16 nm^3^ according to the fold-state crystal structure,^40^ with an estimated refractive index of 1.5867.^41^ Based on mass-to-polarizability calibration for proteins,^42^ we estimate the signal contrast for TG to be approximately 30× lower than that for a 20 nm AuNP, thereby requiring differential imaging for its detection. We specifically performed the assay on the same FOV at different degrees of partial coherence, NA_*i*_ = [0.1, 1.3], keeping the camera counts the same, thereby ensuring equal photon statistics between experiments. By observing the same FOV, we allowed the imaging system to mechanically relax and thus minimized any significant lateral sample drifts. For each imaging condition, the focus position was optimized to yield the maximum absolute signal contrast of TG.

**Figure 6:**
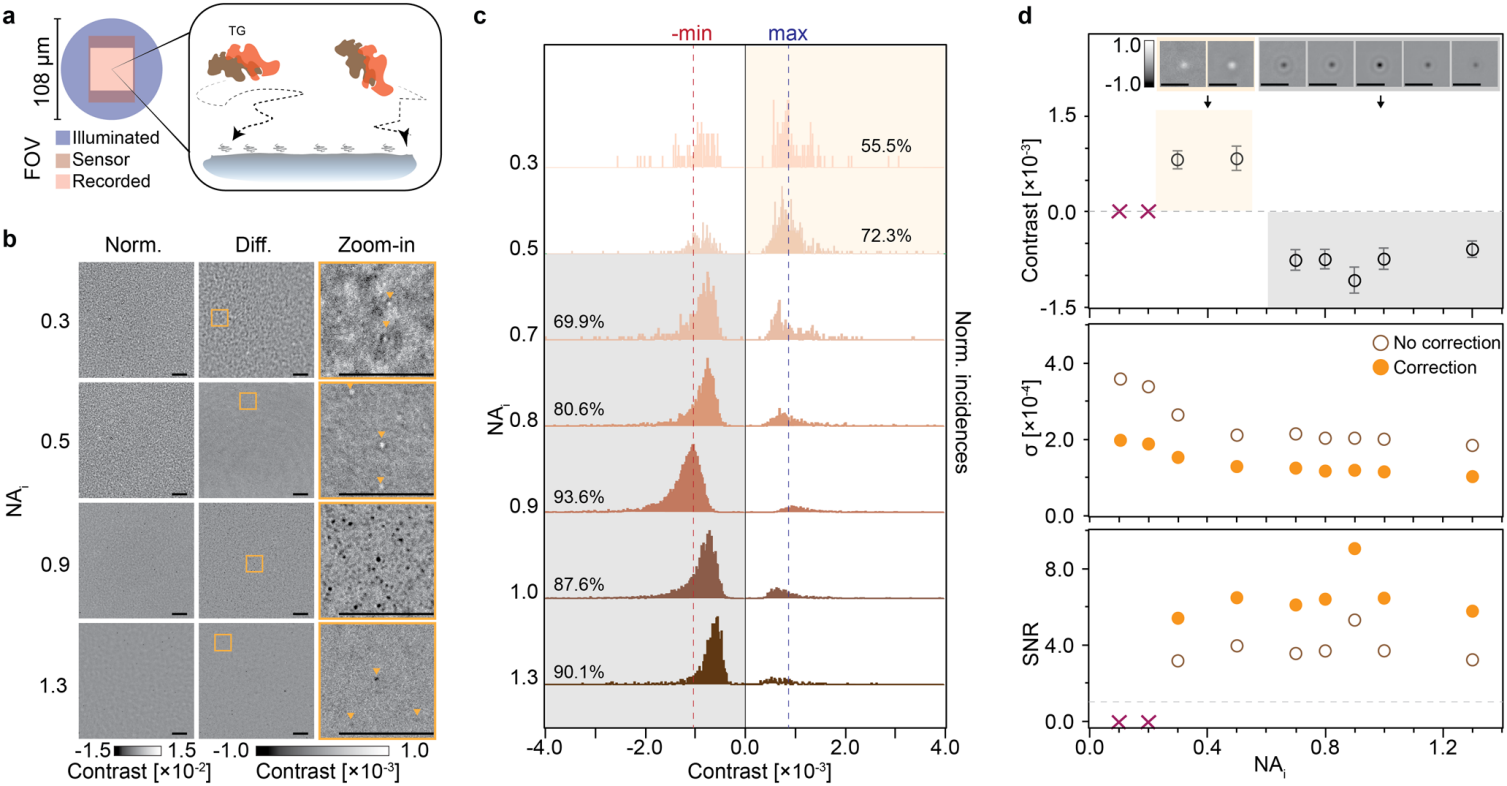
Partial coherence applied to differential imaging: single protein sensitivity. (a) Schematic diagram for large FOV imaging of single thyroglobulin (TG) proteins binding to a glass coverslip via differential imaging. (b) Representative images of single protein binding assays under different degrees of partial coherence. The orange box in the differential column represents the zoomed-in region depicted in the right column. Scale bars: 5 μm. (c) Contrast distribution of the detected particles as a function of the degree of partial coherence. Shaded regions indicate the portion of the distribution attributed to a binding event, along with the percentage of the overall detected particles. The vertical dashed lines indicate the maximum and minimum contrast values of TG across all assays. (d) Corresponding contrast, noise, and SNR of TG as a function of the degree of partial coherence. Noise correction data involves applying a 2D spatial median filter with a kernel size of 55 pixels to reduce residual speckle and beam profile inhomogeneities in the differential images. Inset: ensemble-averaged PSF. Scale bars: 1 μm. Crosses indicate experimental conditions under which it was not possible to detect TG.

Figure 6b shows representative normalized and differential images of the glass surface with TG binding highlighted in the zoomed-in regions. Upon changing from lower degrees of partial coherence to higher ones, a clear contrast signal inversion and reduction in background noise were observed, in agreement with the results from the extracellular vesicle assays. The reduction in noise follows the reduction in speckle contrast contributions that get imprinted into the differential imaging due to the presence of minute sample drifts. All TG binding and unbinding events were subsequently localized, and their respective normalized contrast distribution and fraction of binding events to total particles are plotted in Figure 6c. No particles were detected for NA_*i*_ < 0.3, as they fell below the detection threshold due to a combination of lower signal contrast and higher background noise levels. Differences in the fraction of binding events reflected the increase in false positives attributed to the inclusion of residual speckle contributions with high structural similarity to TG PSFs. Figure 6d summarizes the signal contrast, background noise level, and resulting SNR as a function of the degree of partial coherence for the ensemble of TGs measured, with the inset showing the ensemble-averaged PSFs at each measurement condition. Overall, these data show a trend similar to synthetic and biological NPs, with the lowest SNR at low NA_*i*_, a contrast inversion occurring between NA_*i*_ = 0.5 and 0.7, a maximum contrast around NA_*i*_ = 0.9, followed by a decrease in signal contrast and a monotonic decay in background noise as NA_*i*_ approaches 1.3, where total internal reflection contributions become significant. Lastly, from a signal contrast perspective, the experimental observations are in full agreement with the expected 30-fold reduction in signal going from a 20 nm AuNP (30 × 10^–3^) to a single TG protein (1.4 × 10^–3^).

These results demonstrate that partially coherent systems are also compatible with differential-based imaging and offer SNR tuning similar to that of larger NP systems. Despite the achieved sensitivity being lower compared to specialized label-free protein detection systems, our FOVs are orders of magnitude higher and involve the use of mechanically oscillating RGG systems and cheap laser diodes with poor beam quality. We believe that the sensitivity and throughput can be further improved by engineering the illumination beam profile (see Figure S10), increasing the camera frame rate, and enhancing the speed of the rotating diffuser; the latter two reduce the effects of mechanical drift or time-varying artifacts from the laser and RGG system. As a more suitable solution for differential imaging applications, we propose the use of high-intensity LED systems, as LEDs remove the need for any rotating mechanical elements, offering better stability, suppression of residual time-varying speckles from imperfect RGG synchronization and laser mode hopping, and additional reduction of temporal coherence.^26^

## Conclusion

In this work, we showcased a platform that simultaneously tunes and measures the degree of partial coherence, with the aim of quantifying how this influences the detection sensitivity of single nanoparticles. We further recapitulated the main experimental findings with an imaging model for partially coherent systems. By characterizing the particle signal at different focus positions and the background noise, we demonstrated that a diode laser can achieve performance similar to that of an LED, yet with the advantage of a higher available photon flux. Our results on tuning the spatial coherence over a wide range of synthetic and biological NPs show a consistent optimization of the SNR when the coherence parameter *s* falls within a range of intermediate values, corresponding to 0.7 < NA_*i*_ < 1.3. In the case of biological particles, we verified that single proteins can be enhanced compared with the coherent case due to a synergistic combination of background noise reduction and signal contrast enhancement.

With the defocus scans, we further showed the pivotal role the degree of partial coherence plays in modulating the signal contrast response for different particle types. In the case of imaging systems with a low *s* parameter, the fact that there is no unique focus position that optimizes the contrast for all particle types within heterogeneous NP samples can lead to entire subsets of NPs going undetected when optimizing the contrast for a specific NP population. This highlights the importance of acquiring defocus scans in these imaging systems because they provide valuable information that can be exploited for sizing,^43^ classification,^39^ or sample tilt compensation.^44^ One way to retrieve the axial information, besides time-consuming defocus scans, is to retrieve the phase and perform digital propagation, for instance, by solving the Transport-of-Intensity equation^45^ or conducting these measurements with an off-axis holography configuration. Alternatively, if throughput and sensitivity are paramount, imaging systems with high degrees of partial coherence should be preferred.

Lastly, we have demonstrated that partial coherence imaging is compatible with differential-based detection, thereby promising to increase the throughput of assays, in terms of the total FOV imaged, that rely on the quantification of spatially varying heterogeneous signals that fall below the signal levels of the static background, which can either be in the form of proteins, nucleic acids, lipid nanoparticles, or different charge states. All in all, we believe our work paves the way toward democratizing how inline holographic approaches based on interferometric detection can deliver both high sensitivity and throughput without the need for beam scanning solutions.

## Materials and Methods

### Microscope

The custom-built partially coherent digital holographic optical system was based on a common-path microscope operating in reflection, whereby the illumination and imaging arms were separated by a single 50:50 beamsplitter plate (BSW27, Thorlabs). The setup is outlined in Figure 2e and detailed schematic shown in Figure S6 together with a list of all optical components in Table S1. Partially coherent illumination was achieved by two approaches: focusing a 465 nm laser beam (LDM-465-3000-C, Lasertack) on a rotating ground glass (RGG) diffuser (DG20-1500, Thorlabs) or using a 455 nm LED (M455F3, Thorlabs). For the first option, the laser was coupled out of a single-mode fiber (P1-460A-FC-2, Thorlabs) by a 0.1 NA objective (Olympus UPlan FLN). A plano-convex lens (LA1986, Thorlabs) focused the light on the RGG, which was driven at 600 rpm by a stepper motor (42BYG Stepper Motor, Makeblock). The RGG introduced a power loss of 38%. After the RGG, a 0.4 NA objective (Olympus PlanN) collected the diverging beam. The laser beam with reduced coherence was coupled into a multimode fiber (FT600EMT, Thorlabs) by a 0.3 NA objective (Olympus UPlan FLN) with an overall coupling efficiency of 90%. Both options for partially coherent light sources could be coupled into the inline holography system by a 0.25 NA aspheric lens (C220TMD-A, Thorlabs). A relay system composed of two plano-convex lenses (LA1131 and LA1509-A, Thorlabs) allowed access to the back focal plane, in which an adjustable iris was placed to control the NA_*i*_. The image plane was relay-imaged onto the sample plane via a 3:4 imaging system composed of two plano-convex lenses (AC508-400-A-ML and AC508-300-A, Thorlabs) and a 1.42 oil immersion objective (UPLXAPO 60×, Olympus). The flat-top illumination measured a diameter of 108 μm. In terms of total available photon flux at the same partially coherent parameters for both illumination sources, the RGG system delivered up to 83 mW (8.94 μW/μm^2^) compared to 7.9 mW for the LED (0.85 μW/μm^2^) for the LED system. This represents more than an order of magnitude higher photon flux for the RGG system. The sample plane was relay-imaged using the same objective (UPLXAPO 60×, Olympus) and lens (AC508-300-A, Thorlabs) as those used for illumination. To reduce the area averaged per pixel to 45 × 45 nm, a 50:50 beamsplitter directed a portion of the collected light into a 2:1 relay system composed of two plano-convex lenses (AC508-100-A-ML and AC508-200-A-ML, Thorlabs), which imaged the light onto a CMOS camera (pixel size: 9 μm; BFS-U3-17S7M-C, USB 3.1 Blackfly S, Teledyne). The sensor area was around half the size of the image plane at that position, which restricted the detected FOV to 72 × 50  μm. This imaging system resulted in a 200× magnification. A flip mirror was mounted before the 2:1 relay system, which, when flipped up, guided the light through a plano-convex lens (LA1608-75-A, Thorlabs). Together with the AC508-300-A lens, the last lens relayed images of the back focal plane, which is located inside the objective, onto a second camera (GS3-U3-23S6M, Grasshopper3). The sample focus position was encoded and stabilized using the back reflection from a 670 nm beam (CPS670F, Thorlabs) confocally illuminating the sample. Specifically, the diameter of the reflected beam was used as a feedback parameter in a proportional-integral-derivative loop, making it insensitive to beam-pointing instabilities. The sample was mounted on a motorized XY microstage (Mad City Laboratories) equipped with linear encoders and a Z nanopositioner stage (Nano-Z200, Mad City Laboratories).

### Optical Imaging

During acquisition, a field of view of 46 × 46 *μ*m^2^ corresponding to an area of 1024 × 1024 camera pixels^2^ was recorded with an exposure time of 19.6 ms and a fixed frame rate of 50 Hz. To minimize data load and increase the signal-to-noise ratio, data were saved in the form of 20 time-averaged frames, leading to an effective time resolution of 2.5 Hz. The rotation speed of the diffuser was set to 600 rpm and synchronized with the camera frame rate, such that each effective time-averaged frame would include the average of four revolutions. For all synthetic and biological NP experiments, we measured a power at the sample of approximately 6.5 mW, equivalent to an irradiance of 0.7 μW/μm^2^.

### Sample Preparation

For the experiments with synthetic NPs, we used 142 nm SiO_2_ NPs (SiO_2_-R-L3205-23/1, Microparticles GmbH), 40 nm AuNPs (AuXR40, nanoComposix), and 20 nm AuNPs (EM.STP20, BBI Solutions). All nanoparticles were suspended in deionized water to a concentration of 8 pM for SiO_2_ and 40 nm AuNPs, and 16 pM for 20 nm AuNPs. Before the NP sample was introduced onto the glass surface, each glass coverslip was cleaned with isopropanol and rinsed with deionized water. To locate the approximate focal position, 50 μL of deionized water was first deposited on the coverslip. NPs were then sequentially added by pipetting 1 μL of each stock solution onto the coverslip. After each addition, 5–10 μL of phosphate-buffered saline was introduced to favor nonspecific binding of the NPs to the glass surface due to the reduction of the Debye screening length. The coverslip was then rinsed with deionized water to remove excess particles. Before the measurements were started, an additional 50 μL of deionized water was added to prevent drying during data acquisition.

### EV Isolation and Characterization

The human lung cancer cell line H358 was purchased from the American Type Culture Collection (ATCC: CRL-5807). Cells were cultured in RPMI (ATCC formulation, Gibco A01491) supplemented with 10% fetal bovine serum (FBS, Gibco 10270-106) 1% Pen/Strep (Gibco 15140-122) at 37^◦^C in 5% CO_2_. For EV isolation, cells were first detached with 0.25% Trypsin-EDTA (Gibco, 25200-056), centrifuged at 700 × *g* for 7 min, and the cell pellet was washed with PBS (Gibco, 10010–015). Next, 12× T150 flasks were each plated with 2.5 million H358 cells in 20 mL of RPMI, including 10% EV-depleted FBS (Gibco, A27208-01) and 1% Pen/Strep. After culturing for 72 h to 60–70% confluency, the supernatant was collected and centrifuged at 1500 × *g* for 10 min, then at 10,000 × *g* for 10 min at 4 °C to remove floating cells or large debris. The supernatant was concentrated using an Amicon Ultra-15 centrifugal filter (MWCO = 50 kDa, Merck UFC905096) at 5000 × *g* for 30 min at 4 °C. The concentrated sample was then purified via a size-exclusion chromatography column according to the manufacturer’s specifications (Izon, qEV1 70 nm). Specifically, for each 1 mL of isolated EV sample, 10 mL of PBS were added as the elution volume, from which the first 4.7 mL were discarded and the following 4 mL were collected as the EV fraction. The EV fraction was concentrated with the Amicon Ultra-15 centrifugal filters and afterward supplemented with 1× protease inhibitor cocktail (Thermo Scientific, 87786) before storing at −80 °C until further use.

EVs were lysed in 10× RIPA lysis buffer (Merck 20-188) for Western blot analysis to confirm the characteristic EV biomarkers (CD9, CD63, and TSG101) and the degree of purity using a non-EV marker (GRP94). The blots were probed with the following primary antibodies: anti-CD9 (1:500 dilution, Thermo, 10626D), anti-CD63 (1:1000 dilution, Boster, M01080-1), anti-TSG101 (1:1000 dilution, Biorbyt, ORB1564135), and anti-GRP94 (1:1000 dilution, FineTest, FNab03665). Chemiluminescence was detected using an iBright CL1500 system (Thermo Scientific A44240) with SuperSignal West Pico Plus Chemiluminescence Substrate (Thermo Scientific 34277) and SuperSignal West Atto Ultimate Sensitivity Substrate (Thermo Scientific A38555). The concentration and mean size of the EVs were determined by nanoparticle tracking analysis using Zetaview (Particle Metrix) and found to be 2.8 × 10^10^ particles/mL and 124.4 nm, respectively.

### Image Processing

Each acquired frame was normalized by the median pixel value to correct for shot-to-shot power fluctuations. Sample-independent static contributions were consequently removed by flat-fielding. For this, the median image was computed from 16 different lateral positions at the same focus. Each frame was then divided by the median image computed for the given focus.

### Particle Localization

The first step in particle localization consisted of creating SNR-enhanced images from the normalized flat-field images. For this, the normalized images were binned 2 × 2. Then, the root mean square of the background was computed by including pixels with values smaller than three times the global standard deviation. The standard deviation was estimated from the median absolute deviation. An SNR-enhanced image was created by dividing the binned images by the RMS of the respective background pixels.

All pixels with absolute values larger than 0.2 in the SNR-enhanced image were used as initial guesses for particle localization, provided they additionally fulfilled the requirement of being the local extrema within a 9 × 9 pixel window. These guesses were verified using Trackpy version 0.6.4 and a radial symmetry fit. In the next step, Trackpy version 0.6.4 was used to link the particles at different axial positions. The particle guesses were further considered under the condition that they could be linked along a single trajectory over a distance of 1.5 μm, with a memory of zero and a search range of 1.75.

### Label-Free Detection of Nonspecific Binding of TG

TG (Merck, T1001) was resuspended at 1 mg/mL in water and passed through a 10,000 kDa MWCO filter to remove aggregates. Experiments were performed in a microwell, where a solution of 10 nM TG in PBS was injected prior to imaging. During the acquisition of TG data, a field of view of 46 × 46  μm^2^ corresponding to an area of 1024 × 1024 camera pixels, was recorded with an exposure time of 4.62 ms and a fixed frame rate of 200 Hz. To minimize data load and increase the signal-to-noise ratio, data were saved in the form of 20 time-averaged frames, leading to an effective time resolution of 10 Hz, which was synchronized to the rotation speed of the diffuser to match a single revolution. For all TG experiments, we recorded 500 averaged frames (50 s) with a power at the sample of approximately 26 mW, equivalent to an irradiance of 2.8 μW/μm^2^.

For differential imaging, we computed the rolling differential window average (Δ_*i*_) for the *i*-th frame, *I*_*i*_, as

$$\Delta_{i}=\frac{\overset{N-1}{\underset{j=0}{\sum }}I\left(i+j\right)-\overset{N-1}{\underset{j=0}{\sum }}I\left(i-N+j\right)}{N}$$

with *N* = 50 representing the number of frames averaged. In total, each image in Δ_*i*_ corresponds to effectively averaging 1000 raw camera frames (effective frame rate of 0.2 Hz). For the detection of single binding and unbinding events, no further image processing other than a flat-field correction and a 2D spatial median filter with kernel size *M* = 55 pixels was used to remove illumination inhomogeneities caused by residual speckle, changes in laser mode, and beam-pointing instabilities. Only detection events with track lengths ≥30 time points were considered for further analysis.

## Supplementary Material



## Notes

Model for partially coherent interferometric detection, origin of the contrast enhancement for partially coherent imaging systems, detailed experimental setup and optical components, H358 EV characterization, distribution of maximal contrast of all EVs detected as a function of degree of partial coherence, contrast defocus curves as a function of degree of partial coherence for four EVs, illumination beam engineering enhances the signal contrast (PDF)
