# Supplementary material for: Leveraging Partial Coherence to Enhance Nanoparticle Detection Sensitivity and Throughput in Interferometric Scattering Microscopy
Source: ACS Photonics. 2025 Aug 1;12(8):4376–87. doi: 10.1021/acsphotonics.5c00744 (PMC12371884; doi:10.1021/acsphotonics.5c00744)
Supplement: Supplementary file 1 [file ph5c00744_si_001.pdf]

# Leveraging partial coherence to enhance nanoparticle detection sensitivity and throughput in interferometric scattering microscopy

Chiara Lombardo,[†] Andrea Sottini,[†] Sarina Seiter,[†] Gérard Colas des Francs,[‡] Jaime Ortega Arroyo,[*,†] and Romain Quidant[†]

†*Nanophotonic Systems Laboratory, Department of Mechanical and Process Engineering, ETH Zurich, Tannenstrasse 3, 8092 Zurich, Switzerland*

‡*Université Bourgogne Europe, CNRS, Laboratoire Interdisciplinaire Carnot de Bourgogne ICB UMR 6303, F-21000 Dijon, France*

E-mail: jarroyo@ethz.ch

Number of pages: 23

Number of figures: 10

Number of tables: 1

# 1 Model for partially coherent interferometric detection

To gain better insight into our system we developed an imaging model based on partial coherence that recapitulates the experimental contrast defocus curves shown in Fig. 4 of the main text. This model builds on existing results from literature, and is divided into three sections, whereby we specifically mention the approximations that can be made in the current configuration: i) the excitation and reflected field considering incoherent Köhler illumination, ii) dipolar scattering in the objective and iii) optical path aberration appearing in defocused interferometric microscopy.

**Incident and reflected fields** We follow ref.[1] and model the Köhler illumination considering incoherent plane waves impinging on the glass coverslip interface at angles $(\theta_m, \phi_m)$ that span the numerical aperture of an oil immersion objective. The objective images the focus $(z = 0)$ given a specified glass substrate (optical index $n_g$, thickness $t_g$) and oil immersion film (optical index $n_i$, thickness $t_i$), see Fig. S1. Deviations from optimal conditions will be introduced later using an aberration phase shift. We denote $r_s, r_p$ $(t_s, t_p)$ as the the coverslip Fresnel coefficient of reflection (transmission) for the TE and TM polarisations, respectively. Note that the coverslip Fresnel coefficients are approximated for small oil/glass optical contrast, and the phase shift originating from the oil and glass thickness will be included in the aberration phase shift.[2] Using the angular spectrum representation[3] we express the incident transmitted field that excites the nanoparticle as:

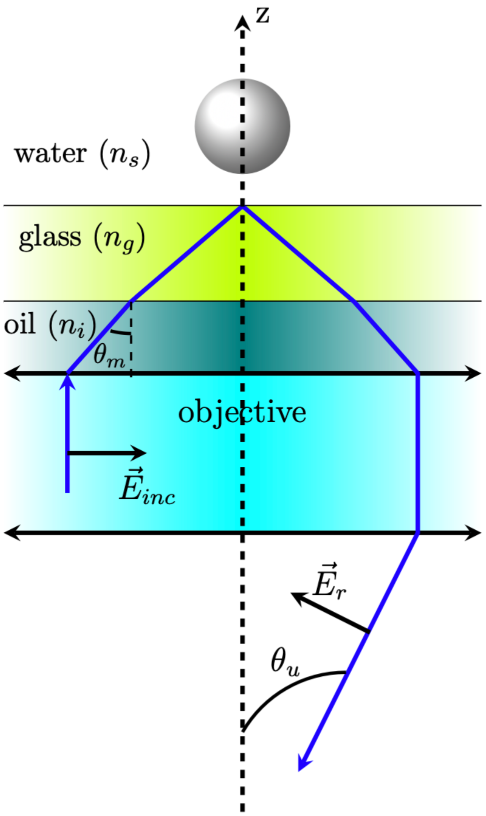


Figure S1: Plane wave expansion model for incoherent Köhler illumination. Each plane wave is characterised by an incident angle $\theta_m$, which reflect on the coverslip and is finally detected at the image plane of the objective at the output angle $\theta_u$.

$$\vec{E}_t(O) = A_1 E_0 \sin\theta_m \sqrt{\cos\theta_m} \begin{pmatrix} t_p \cos^2\phi_m \cos\theta_s + t_s \sin^2\phi_m \\ (t_p \cos\theta_s - t_s)\cos\phi_m \sin\phi_m \\ -t_p \cos\phi_m \sin\theta_s \end{pmatrix} \quad (1)$$

$$\cos\theta_s = \sqrt{1 - \frac{n_i^2}{n_s^2}\sin^2\theta_m}$$

In the following, we note $w_s = n_s k_0 \cos\theta_s$ the vertical component of the wavector in water with the angle in water following the Snell-Descartes law $n_s \sin\theta_s = n_i \sin\theta_m$. Similar definitions occurs in all media. The coverslip Fresnel coefficients are approximated for small

oil/glass optical contrast. Indeed, one can then write

$$t^{slab} = \frac{t_{o/g} t_{g/w} e^{iw_g t_g}}{1 + r_{o/g} r_{g/w} e^{2iw_g t_g}} e^{i(w_i z_i - w_s z_s)} \approx t_{o/g} t_{g/w} e^{i\Psi_t}$$

where $\Psi_t$ is a phase shift that will be rewritten as an aberration optical path to describe the deviation from the design configuration.[2] So that $t_{s/p} = t_{o/g}^{s/p} t_{g/w}^{s/p}$ has to be used in the above expressions.

In adddition, the reflected field imaged at the position of the detector writes:

$$\vec{E}_r(O') \approx \frac{A_2}{M^2} E_0 \sin\theta_m \cos\theta_m \begin{pmatrix} -r_p \cos^2\phi_m + r_s \sin^2\phi_m \\ -(r_p + r_s)\cos\phi_m \sin\phi_m \\ 0 \end{pmatrix}$$

Again, the Fresnel coefficient are approximated for small oil/glass optical contrast.

$$r^{slab} = \frac{r_{o/g} + r_{g/w} e^{2iw_g t_g}}{1 + r_{g/w} e^{2iw_g t_g}} e^{-2iw_i t_g} \approx r_{g/w} e^{i\Psi_r}$$

$\Psi_r$ is a phase shift that will included also in the an aberration optical path. $r_{s/p} = r_{g/w}^{s/p}$ has to be used.

In the above equations, focusing parameters associated to lens 1 and lens 2 are written as: $A_1 = ik_0 f_1 e^{-in_i 0 k_0 f_1}/2\pi$ and $A_2 = ik_0 f_2 e^{-ik_0 f_2}/2\pi$. In this description, we have used the approximations $\sin\theta_u = \sin\theta/M \ll 1$ and $\cos\theta_u \approx 1$ based on the large magnification $M$ of the microscope.

**Scattered field** We approximate the scattered field from the NP as that of an induced dipole; namely:

$$\vec{p} = \alpha_p \vec{E}_t(O)\ ; \alpha_p = 4\pi R^3 \epsilon_p \frac{\epsilon_p - \epsilon_s}{\epsilon_p + 2\epsilon_s}. \quad (2)$$

The dipolar electric field radiated by the dipolar source is determined from the angular representation $\vec{E}_o$. For a particle far from the glass/water interface, one can use the approximation[4,5] $\vec{E}_o = \left[(\vec{p} \cdot \vec{e}_{\parallel})\vec{e}_{\parallel} + (\vec{p} \cdot \vec{e}_{\perp})\vec{e}_{\perp}\right]$ with $\vec{e}_{\parallel,\perp}$ TE/TM polarized unit vectors. However, for a particle on the substrate, the evanescent coupling cannot be neglected and the exact angular representation has to be considered.[1,6] We obtain at the detector position $O' = (0, 0, 0)$ in the image plane, again considering approximation for large microscope magnification $M$:

$$\vec{E}_s \approx I_0 \left(p_x\vec{e}_x + p_y\vec{e}_y\right)$$
$$I_0 \approx \frac{in_i^{3/2}k_0^3}{n_sM^2}\frac{A_2}{2A_1}\int_0^{\theta_{det}}\left(\tau_p + \frac{k_s}{w_s}\tau_s\right)(\cos\theta)^{\frac{3}{2}}\sin\theta\mathrm{d}\theta$$

with $\tau_{s,p}^{slab}$ the Fresnel coefficients of transmission of the coverslip from water to oil (again approximated for small oil glass optical contrast). As an approximation, we assume a point-like detection at $O'$ and thus consider the scattering contribution of vertical dipole negligible for large magnification.[3] However, for a fully comprehensive model, the signal should be integrated over the sensor area, as the field scattered by a vertical dipole would also contribute to the detected signal (see also the discussion in §2.3).

**Aberration optical path** Under ideal conditions, oil immersion objectives are designed to focus at the glass/water interface when the specific glass substrate (optical index $n_{g0}$, thickness $t_{g0}$) and oil immersion film (optical index $n_{i0}$, thickness $t_{i0}$) are met. However, in practice, the glass substrate can have small deviations in $n_g$ and $t_g$; thus, the oil immersion layer has to be adapted accordingly (optical index $n_i$ , thickness $t_i$). Moreover spherical aberration affects the position of the focus. To account for spherical aberration, we apply a correction factor $\Delta_f$ between the movement of the focal plane $z_f$ and the movement of the objective $z_{exp}$: $z_f = \Delta_f z_{exp}$ with[7]

$$\Delta_f = \frac{\tan\left(\sin^{-1}(0.5\mathrm{NA_{obj}}/n_i)\right)}{\tan\left(\sin^{-1}(0.5\mathrm{NA_{obj}}/n_s)\right)}$$

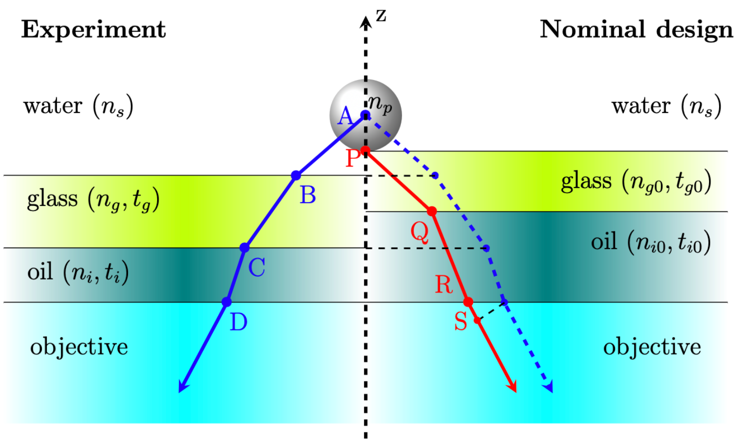


Figure S2: Optical rays description of the aberration introduced for a microscope objective working in actual (left) or design (right) conditions.

We can write the thickness of the oil immersion layer as a function of the other system parameters as:[2]

$$t_i = z_p - z_f + n_i \left( \frac{t_{g0}}{n_{g0}} - \frac{t_g}{n_g} + \frac{t_{i0}}{n_{i0}} - \frac{z_p}{n_s} \right). \tag{3}$$

The optical path difference for the scattered dipolar field $\Lambda_s = \Lambda(\theta) = (PQRS) - (ABCD)$ between the experiment and nominal design follows as (see also Fig. S2):

$$\begin{aligned} \Lambda(\theta) = \quad & z_p \sqrt{n_s^2 - n_i^2 \sin^2 \theta} + n_i \cos \theta t_i - t_{i0} \sqrt{n_{i0}^2 - n_i^2 \sin^2 \theta} \\ & + t_g \sqrt{n_g^2 - n_i^2 \sin^2 \theta} - t_{g0} \sqrt{n_{g0}^2 - n_i^2 \sin^2 \theta} \end{aligned} \tag{4}$$

Optical path aberrations for the reflected and transmitted fields at angle $\theta_m$ are accounted by including the following expressions:

$$\begin{aligned} \Lambda_r(\theta_m) &= 2 \left( n_i t_i \cos \theta_m - t_{i0} \sqrt{n_{i0}^2 - n_i^2 \sin^2 \theta_m} + t_g \sqrt{n_g^2 - n_i^2 \sin^2 \theta_m} - t_{g0} \sqrt{n_{g0}^2 - n_i^2 \sin^2 \theta_m} \right) \\ \Lambda_t(\theta_m) &= z_p \sqrt{n_s^2 - n_i^2 \sin^2 \theta_m} + n_i t_i \cos \theta_m - t_{i0} \sqrt{n_{i0}^2 - n_i^2 \sin^2 \theta_m} \\ & \quad + t_g \sqrt{n_g^2 - n_i^2 \sin^2 \theta_m} - t_{g0} \sqrt{n_{g0}^2 - n_i^2 \sin^2 \theta_m} \end{aligned}$$

Given that the detected intensity depends on the phase difference between the scattered and reference fields; the scattered field through the objective is updated including a phase shift $\Lambda_{tot} = \Lambda_s(\theta) + \Lambda_t(\theta_m) - \Lambda_r(\theta_m)$. That is

$$
\begin{aligned}
I_0 &\rightarrow \frac{i n_i^{3/2} k_0^3}{n_s M^2} \frac{A_2}{2A_1} e^{ik_0 \Lambda_m(\theta_m)} \int_0^{\theta_{det}} \left( \tau_p + \frac{k_s}{w_s} \tau_s \right) e^{ik_0 \Lambda(\theta)} (\cos\theta)^{\frac{3}{2}} \sin\theta \mathrm{d}\theta \\
\Lambda_m(\theta_m) &= \Lambda_t(\theta_m) - \Lambda_r(\theta_m) \\
&= z_p \sqrt{n_s^2 - n_i^2 \sin^2\theta_m} \\
&\quad -(n_i t_i \cos\theta_m - t_{i0}\sqrt{n_{i0}^2 - n_i^2 \sin^2\theta_m} + t_g \sqrt{n_g^2 - n_i^2 \sin^2\theta_m} - t_{g0}\sqrt{n_{g0}^2 - n_i^2 \sin^2\theta_m})
\end{aligned}
$$

**Numerically computed contrast signal** Finally, we reconstruct the contrast signal from the detected intensity as the incoherent sum of the reflected plus scattered fields resulting from the Köhler excitation within the illumination numerical aperture $\mathrm{NA_i} = n_i \sin\theta_m^{max}$

$$
I_{det} = \sum_{\theta_m \leq \theta_m^{max}} |\vec{E}_r(\theta_m) + \gamma \vec{E}_s|^2 \tag{5}
$$

We add an additional factor $\gamma$ as function of $\mathrm{NA}_i$ to take into account detection efficiency of the imaging system together with discrepancies between the model and experiment in the form of differences in the beam illumination profile at the BFP of the objective, which impact the effective reflectivity (§2.1), differences in the effective scattering cross-section due to the increase in resolution (§2.2), and deviations in the scattering contributions from the dipole approximation depending on the particle size and material (§2.3).

**Results** Fig. S3 (a-c) show the experimental results of the detected 20nm AuNP, 40nm AuNP and 142 nm $SiO_2$ contrast as a function of the axial defocus for different coherence parameters. The solid curves with shaded area correspond to the ensemble average ± one standard deviation, as shown in the main text Fig. 4. The overlaid brown curves correspond to a global fit to the partially coherent imaging model for all three particles and over the different degrees of partial coherence at once. With the exception of some high-frequency

oscillations away from the focus, the experimental results show excellent agreement with the imaging model within $\pm$ one standard deviation of the ensemble average. We attribute this discrepancy to the spatial pixel averaging used to determine the average experimental contrast in the defocus curves. Our experimental results together with the simulations demonstrate that increasing the degree of partial coherence enhances the contrast of scattering particles. This trend is partially captured by the fit parameter $\gamma$ (Eq. 5), whose function is to compensate for the limitations in the model with respect to the experiment (Fig. S3d).

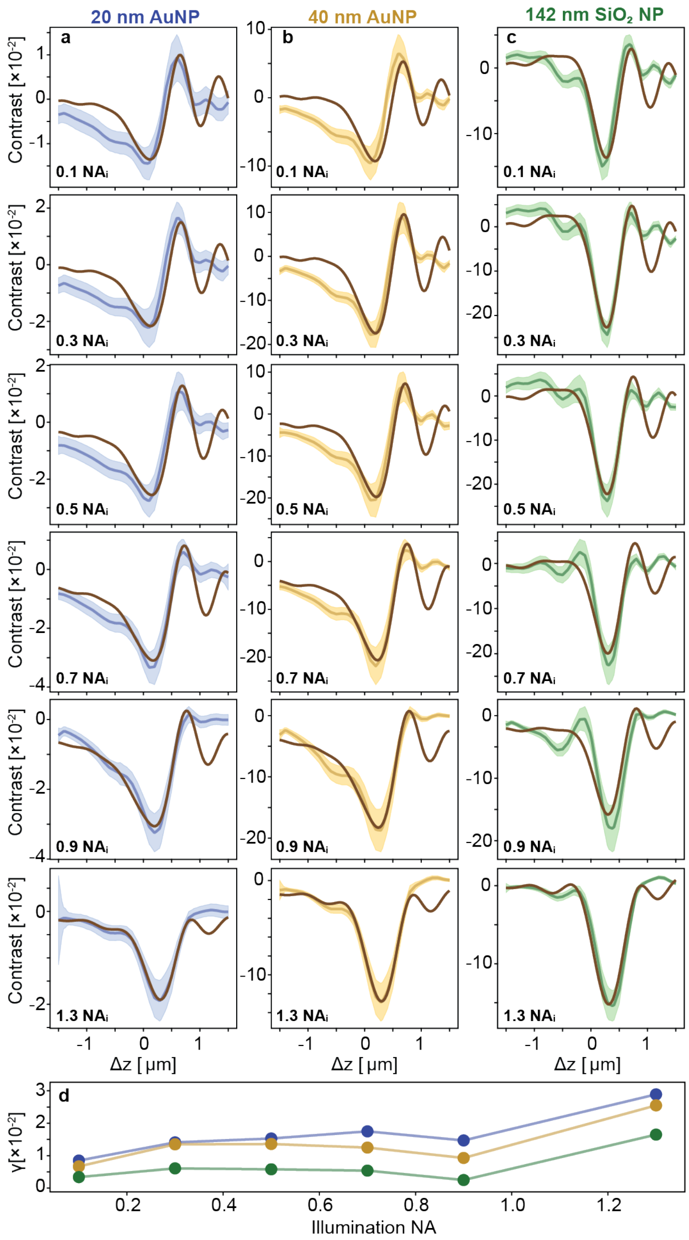


Figure S3: Partially coherent imaging model for individual NPs as a function of defocus. (a-c) Contrast defocus curves as a function of $NA_i$ for 20 nm AuNP, 40 nm AuNP and 142 nm $SiO_2$ NPs, respectively. Solid curves with shaded area correspond to the experimental NP ensemble average ± one standard deviation. Overlaid brown curves correspond to fits to the partially coherent imaging model. (d) Scattering field amplitude correction for the different NPs and illumination NAs. Nominal parameters are $n_{g0} = 1.5$, $t_{g0} = 170\,\mu m$, $n_{i0} = 1.5$, $t_{i0} = 100\,\mu m$. Fitting parameters common to all measurement are $n_g = 1.502$, $t_g = 174\,\mu m$, $n_i = 1.5007$. An axial offset $z_o = -71$ nm is also applied to all fitting curves.

# 2 Origin of the contrast enhancement in a partially coherent imaging system

To understand the complex dependence of the signal contrast on the degree of partial coherence, we decomposed the main contributing parameters for an interferometric microscope configured in a reflection geometry. For sake of simplicity, we isolated how partial coherence affects each parameter individually, whilst keeping the rest fixed. In terms of parameters, we only considered the reflectance, the imaging resolution, and the scattering emission profile under the dipole approximation.

Given that we only want to outline the general trends rather than perform a comprehensive study, we have excluded any effects due to differences in phase transfer function, differences in phase between polarisation states, optical aberrations, and additional reflections from other interfaces in the imaging system.

## 2.1 Effect of reflectance on the scattering contrast scaling as a function of degree of partial coherence

To determine whether the difference in signal contrast results from the change in reflectance, $R$, as the degree of partial coherence increases, we analysed its effect in the absence of any other contributions. To derive the dependence of contrast on $R$, $C(R)$, we first express the measured intensity from an interferometric-based detection as:

$$I_{det} \propto |E_R + E_s|^2 = |E_i|^2(R + \sigma + 2\sqrt{R}\sqrt{\sigma}\cos\theta), \tag{6}$$

with $E_R$ and $E_s$ being the reflected and scattered field, $\sigma$ the scattering cross-section of the nanoparticles, and $\theta$ the phase difference. Assuming that for all NPs considered in this work, $\sigma$ is much smaller than $R$ for the glass-water interface, and normalising the detected

intensity by $E_R^2$, we can now express the measured contrast as:

$$C(R) = \frac{2}{\sqrt{R}}\sqrt{\sigma}\cos\phi. \tag{7}$$

The above expressions shows that the amount of light reflected at the coverslip-medium interface directly influences the contrast. Given that $R$ is a function of the incoming angle, the signal contrast depends on the $\mathrm{NA}_i$ and thus the coherence parameter. Fig. S4 shows how the reflectance changes for increasing coherence parameter. The first row shows the simulated case for flat top illumination in the backfocal plane, as shown in Fig. S4(a). Column (b) shows the reflectance when sampling the $\mathrm{NA}_i$ as slices corresponding to single k-vectors of increasing size. In this case, the reflectance rapidly rises at the $\mathrm{NA}_i$ corresponding to the critical angle and drops at the NA limits of the objective, in agreement with Fresnel coefficients derived for the glass-water interface. Column (c) shows the more experimentally relevant case of sampling the $\mathrm{NA}_i$ as circular areas of increasing radii. In this case, $R_{norm}$ describes the average reflectance upon including all k-vectors contributions inside the given BFP area. As in the prior case, the reflectance increases when the $\mathrm{NA}_i$ approaches the critical angle, although to a smaller extent. Assuming all other variables remain constant in Eq. 7, the experimental contrast can be estimated by taking the inverse square root of $R_{norm}$, as shown in (d). The decay in contrast for increasing $\mathrm{NA}_i$ coincides with the experimental results of prior work.[8–10]

In contrast to prior work, our platform relay images the flat top illumination from the MMF to the object plane, which results in a sinc-shaped illumination profile at the BFP. Consequently, the reflectance measured experimentally from the relay-imaged BFP is composed of the superposition of the beam profile and the reflectance from the glass-water interface derived in the first row. This is evident in (b) and (c), where $R$ rapidly rises at the critical angle but slightly decreases at larger $\mathrm{NA}_i$ values. As a result of the sinc-shaded profile, the higher angular illumination components, which inherently have higher reflectance

values, are weighed less compared to the flat-top illumination. This suppresses the decrease in contrast due to higher effective reflectance, whilst simultaneously decreasing coherent artifacts. Moreover, our experimental configuration leads to an overall contrast enhancement as the $NA_i$ increases, with a maximum occuring at 1.2, a reversal of the trend observed when illuminating a flat-top illumination profile at the BFP.

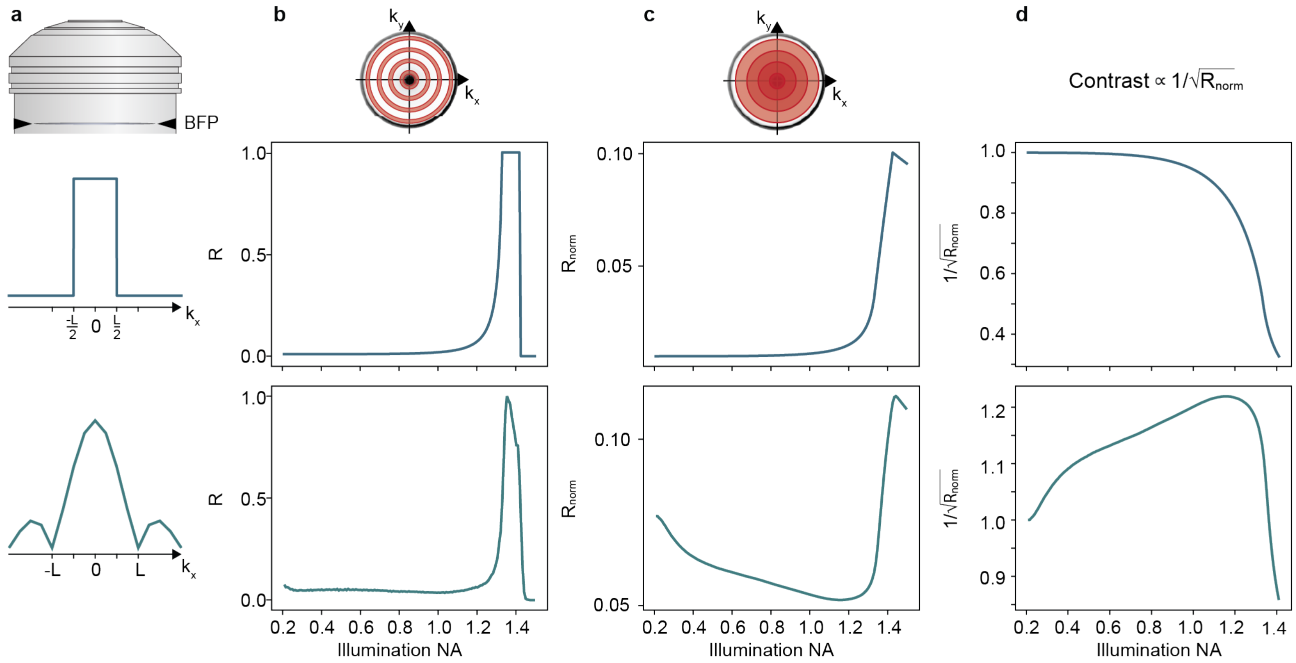


Figure S4: Effect of partial coherent illumination on the effective reflectance from the glass water interface. (a) Schematic illustrating two different implementations of partial coherent illuminations by shaping the beam profile at the BFP. Middle row: simulated data resulting from a top hat illumination; bottom column: experimentally retrieved data from the sinc-like illumination profile at the BFP. (b) Reflectance from the water/glass interface within each spatial frequency, retrieved from integrating the power at each ring from the BFP image, expressed as a function of $NA_i$. (c) Effective reflectance from the water/glass interface over the total illuminated spatial frequency space expressed as a function of $NA_i$. (d) Effective contrast scaling caused by changes in the effective reflectance as a function of $NA_i$.

## 2.2 Effect of resolution increase on the scattering contrast scaling as a function of degree of partial coherence

To determine whether the difference in signal contrast results from the increase in resolution as the degree of partial coherence increases, it suffices to express the partial coherent

diffraction limit as $(1+s)^{-1}\rho$, where $\rho = \lambda/NA$, which is derived under the assumption of weak object optical transfer function – meaning the absorption and phase of the NPs are sufficiently small. This leads to the well-known expressions of the diffraction limit for the coherent ($\rho$) and incoherent ($\rho/2$) cases. Experimentally we can assume that the full-width-at-half-maximum of the diffraction limit as a measure of $(1+s)^{-1}\rho$, so the diffraction limited area as a function of degree of partial coherence can be expressed as $A(s) = \pi(1+s)^{-2}\rho^2/4$. If so, we can describe the amount of scattered photons in this area per unit time as:

$$P_s(s) = \gamma \frac{\sigma}{A(s)} P_i \tag{8}$$

where $\gamma$, and $P_i$ correspond to the collection efficiency of the microscope, and the incident number of photons per area per unit time.[11] If we then normalise the total amount of photons with respect to the coherent case we arrived at the following expression:

$$\frac{P_s(s)}{P_s(0)} = (1+s)^2. \tag{9}$$

However, because the contrast signal results from an interferometric-based detection, we can approximate the number of photons in this term as $P_{int} = 2\sqrt{P_s P_r}$. Assuming that the effective reflectivity of the glass-water interface remains the same, and the degree of partial coherence does not influence any other parameters, the expected contrast signal enhancement from an increase in resolution depends linearly on $(1+s)$, i.e. the degree of partial coherence. These back-of-the-envelope calculations lead to an up to a two-fold increase in contrast caused by the increase in resolution.

## 2.3 Dipole orientation and scattering efficiency as a function of degree of partial coherence

To determine whether the difference in signal contrast is affected by the collection efficiency caused by the change in dipole excitation from low to high NA of illumination,[8] we simu-

lated the scattering emission profile of different particles at the glass-water interface under the dipole approximation (Figure S5). Specifically, the different particle species were approximated as point sources positioned at a distance equal to the particle's radius away from the interface. For small $NA_i$s, corresponding to a low degree of partial coherence, we assumed that the incident light preferentially excites dipoles parallel to the interface in the NPs, represented by a horizontal dipole. In contrast, for larger $NA_i$s, the incident light composed of higher spatial frequencies leads to excitation of partially vertical dipoles in the NPs, represented in the plots as a vertical dipole.

The upper row of plots in Fig. S5 shows the backwards (-90° to 90°) and forwards (-180° to -90° and 90° to 180°) scattering of the three particles, under either a perfectly horizontal (left) or vertical (right) dipole orientation, respectively. The lower row shows the corresponding backwards scattering contribution, with the gray dashed lines delineating the maximum collection angle from the detection objective (1.42 NA, oil immersion). Despite that for the vertical dipole case, a larger portion of light is backscattered compared to the horizontal dipole case; the total scattered intensity is lower for vertical dipoles, resulting in a decrease in collected light of 11%, 13%, and 19% for 20 nm AuNPs, 40 nm AuNPs, and 142 nm $SiO_2$ NPs, respectively, compared to the horizontal dipole case. This trend of decreasing scattering intensity, and thus, lower contrast as a function of degree of partial coherence, was not observed experimentally. As such, we can rule out this contribution as being the predominant factor affecting the contrast modulation. Furthermore, even at the highest $NA_i$ examined, a combination of horizontal and vertical dipoles is present, rather than purely vertical dipole contribution. Lastly, describing the emission as a pure dipole point source is not completely accurate, particularly for the 142 nm silica particles, where higher-order modes become increasingly relevant.

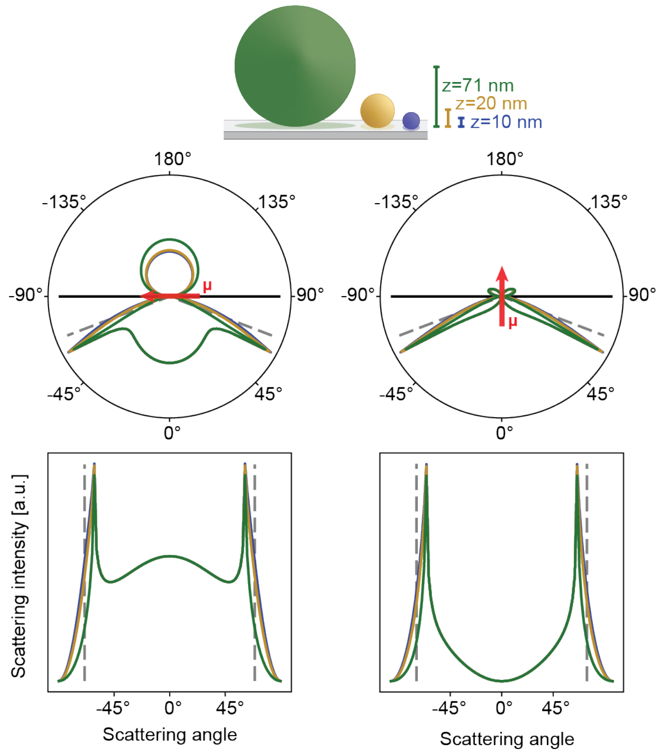


Figure S5: Effect of partial coherent illumination on scattering emission profile. Scattering emission profile for a 2D dipole located on the glass-water interface 20 nm AuNPs, 40 nm AuNPs, 143 nm $SiO_2$ NPs illuminated at low (left) and high (right) degrees of partial coherence. Dashed vertical lines represent the angle of collection denoted by the 1.42 NA detection objective.

# 3 Experimental setup

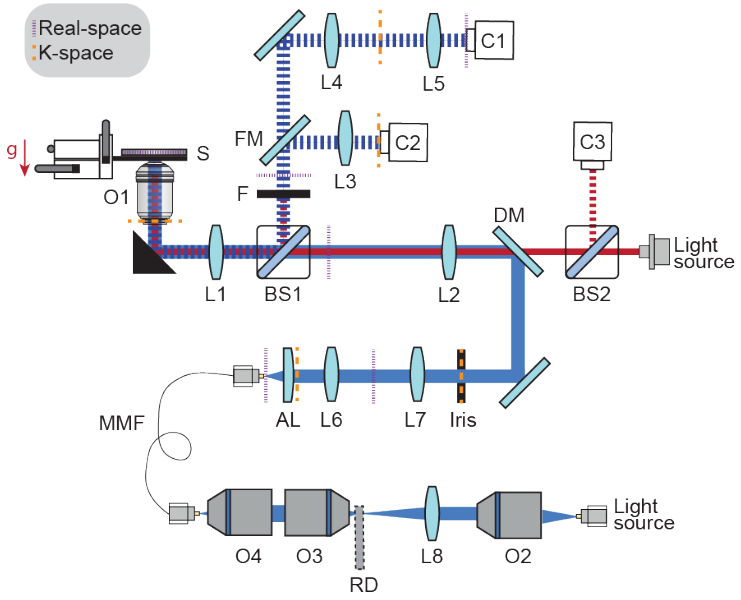


Figure S6: Custom-built partially coherent digital holographic optical system. The pruple and orange dashed lines indicate the position of the real- and k-space. The system is composed of four objectives (O), seven lenses (L), three cameras (C), two beamsplitters (BM), a shortpass filer (F), a dichroic mirror (DM), an aspheric lens (AL), a flip mirror (FM) and a rotating diffuser (RD). All component specifications are given in Tab. S1.

Table S1: Component specifications of the custom-built partially coherent digital holographic optical system.

| **Label** | **Description** | **Part Number / Model** |
|---|---|---|
| Imaging laser | 465 nm diode laser module | LDM-465-3000-C (LaserTack) |
| Autofocus laser | 670 nm diode laser module | CPS670F (Thorlabs) |
| LED | 455 nm LED | M455F3 (Thorlabs) |
| O1 | 60x/1.42 Oil, Infinity | UPLXAPO |
| O2 | 4x/0.10, Infinity | Olympus Uplan FLN |
| O3 | 20x/0.40, Infinity | Olympus PlanN |
| O4 | 10x/0.30, Infinity | Olympus Uplan FLN |
| AL | f = 11 mm, NA = 0.25 | C220TMD-A (Thorlabs) |
| C1 | 1600x1100, 9 µm pixel, 196 fps | BFS-U3-17S7M-C (Blackfly S) |
| C2 | 1920x1200, 5.86 µm pixel, 163 fps | GS3-U3-23S6M (Grasshopper3) |
| C3 | 1280x1024, 5.2 µm pixel, 30 fps | DCC1545M (Thorlabs) |
| L1 | f = 300 mm | AC508-300-A (Thorlabs) |
| L2 | f = 400 mm | AC508-400-A-ML (Thorlabs) |
| L3 | f = 75 mm | LA1608-75-A (Thorlabs) |
| L4 | f = 100 mm | AC508-100-A-ML (Thorlabs) |
| L5 | f = 200 mm | ACT508-200-A-ML (Thorlabs) |
| L6 | f = 50 mm | LA1131 (Thorlabs) |
| L7 | f = 100 mm | LA1509 A (Thorlabs) |
| L8 | f = 125 mm | LA1986 (Thorlabs)(Thorlabs) |
| BS1 | 50:50 beamsplitter | BSW27 (Thorlabs) |
| BS2 | 50:50 beamsplitter cube | BS016 (Thorlabs) |
| DM | Dichroic mirror | – |
| SMF | NA = 0.13, 1 m | P1-460A-FC-2 (Thorlabs) |
| MMF | 600 µm core diameter | FT600EMT (Thorlabs) |
| F | 650 nm shortpass filter | FES0650 (Thorlabs) |
| RGG | 1500 grit, Ø2” | DG20-1500 (Thorlabs) |

## 4 Supplementary figures for EV-based measurements

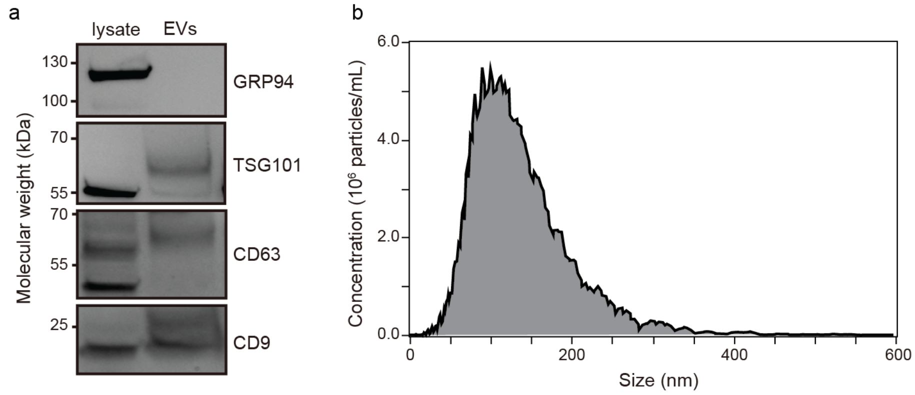


Figure S7: H358 EV characterisation. (a) Western blot analysis against specific (TSG101, CD63, CD9) and non-specific (GRP94) EV markers. (b) Size and concentration determination via nanoparticle tracking analysis.

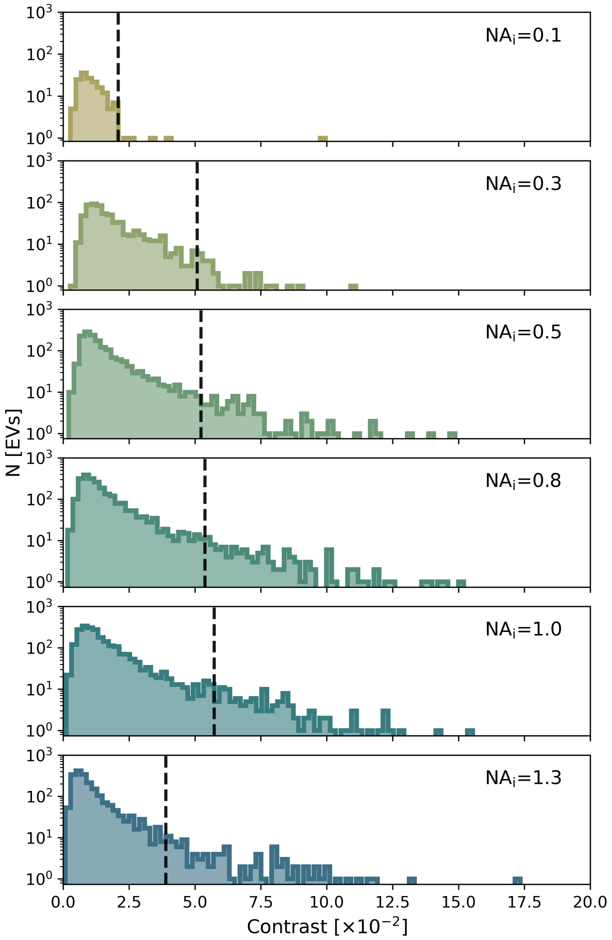


Figure S8: Distribution of the maximal contrast of all detected EVs as a function of the degree of partial coherence. Dashed lines indicate the 95th percentile value. Increasing from the smallest to the largest $\mathrm{NA}_i$, the counts of considered EVs are 160, 683, 1746, 2501, 2491, 2098.

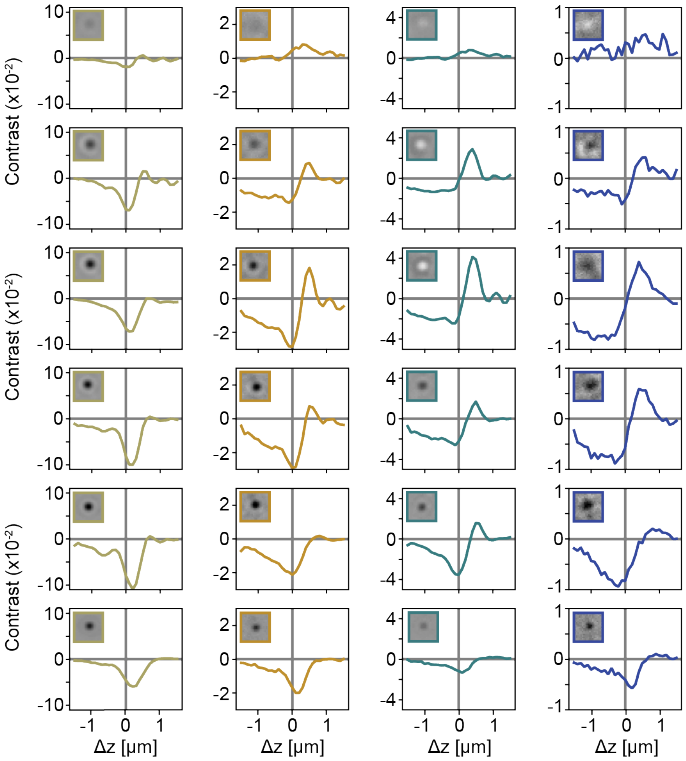


Figure S9: Contrast defocus curves as a function of the degree of partial coherence for the four representative EV particles shown in Figure 5b-d. Inset: zoom-in image of the PSF of the EVs at the focus position that maximises the absolute signal contrast value.

# 5 Supplementary figure for single protein sensing and illumination beam engineering

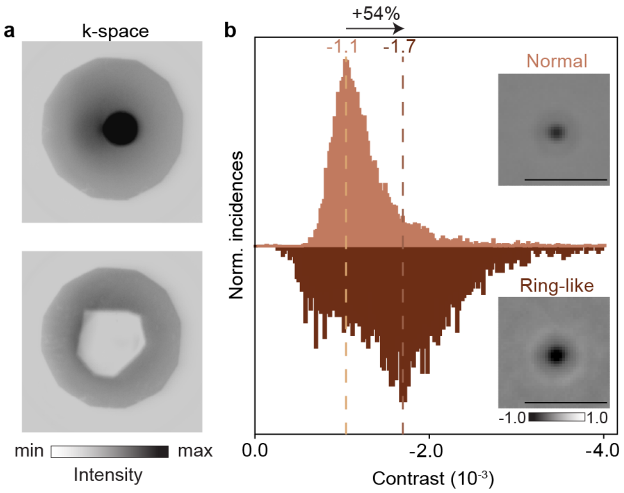


Figure S10: Illumination beam engineering enhances the signal contrast. (a) BFP image upon a partial coherent illumination corresponding to $NA_i$=0.9 without (top) and with (bottom) illumination beam engineering. For beam profile engineering, an additional aperture stop was placed in the illumination module of the setup to create a ring-like partially coherent illumination profile. (b) Corresponding contrast distribution of the detected TG binding events comparing both illumination profiles, with vertical dotted lines indicating the maxima of each distribution. The ring-like illumination leads to a 54% increase in contrast signal from which a second weaker population is discernible, corresponding to the TG monomer. Inset: ensemble-averaged particle PSF for all binding events. Scale bar: 1 µm

# References


(1) Avci, O.; Adato, R.; Ozkumur, A. Y.; Ünlü, M. S. Physical modeling of interference enhanced imaging and characterization of single nanoparticles. *Opt. Express* **2016**, *24*, 6094–6114.

(2) Haeberlé, O. Focusing of light through a stratified medium: a practical approach for computing microscpoe point spread functions/ Part I: conventional microscopy. *Optics Commun.* **2003**, *216*, 55–63.

(3) Novotny, L.; Hecht, B. In *Principles of Nano-Optics*; Press, C. U., Ed.; Cambridge University Press, 2012.

(4) Aguet, F.; Geissbuhler, S.; Marki, I.; Lasser, T.; Unser, M. Super-resolution orientation estimation and localization of fluorescent dipolesusing 3-D steerable filter. *Opt. Express* **2009**, *17*, 6829.

(5) Dong, J.; Maestre, D.; abd T. Juffmann, C. C.-B. Fundamental bounds on the precision of iSCAT, COBRI and dark-field microscopy for 3D localization and mass photometry. *J. Phys. D: Appl. Phys.* **2021**, *54*, 394002.

(6) Khadir, S.; Chaumet, P.; Baffou, G.; Sentenac, A. Quantitative model of the image of a radiating dipole through a microscope. *JOSA A* **2019**, *36*, 478.

(7) Diel, E.; Lichtman, J.; Richardson, D. Tutorial: avoiding and correcting sample-induced spherical aberration artifacts in 3D fluorescence microscopy. *Nat. Protoc.* **2020**, *15*, 2773.

(8) Avci, O.; Campana, M. I.; Yurdakul, C.; Ünlü, S. Pupil function engineering for enhanced nanoparticle visibility in wide-field interferometric microscopy. *Optica* **2017**, *4*, 247–254.

(9) Mazaheri, M.; Kasaian, K.; Albrecht, D.; Renger, J.; Utikal, T.; Holler, C.; Sandoghdar, V. iSCAT microscopy and particle tracking with tailored spatial coherence. *Optica* **2024**, *11*, 1030–1038.

(10) Wu, B. K.; Tsai, S. F.; Hsieh, C. L. Simplified Interferometric Scattering Microscopy Using Low-Coherence Light for Enhanced Nanoparticle and Cellular Imaging. *J. Phys. Chem. C* **2025**, 5075–5085.

(11) Zuo, C.; Li, J.; Sun, J.; Fan, Y.; Zhang, J.; Lu, L.; Zhang, R.; Wang, B.; Huang, L.;

Chen, Q. Transport of intensity equation: a tutorial. *Opt. Lasers Eng.* **2020**, *135*, 106187.
